# Structural Insights into the Intracellular Region of the Human Magnesium Transport Mediator CNNM4

**DOI:** 10.3390/ijms20246279

**Published:** 2019-12-12

**Authors:** Paula Giménez-Mascarell, Iker Oyenarte, Irene González-Recio, Carmen Fernández-Rodríguez, María Ángeles Corral-Rodríguez, Igone Campos-Zarraga, Jorge Simón, Elie Kostantin, Serge Hardy, Antonio Díaz Quintana, Mara Zubillaga Lizeaga, Nekane Merino, Tammo Diercks, Francisco J. Blanco, Irene Díaz Moreno, María Luz Martínez-Chantar, Michel L. Tremblay, Dominik Müller, Dritan Siliqi, Luis Alfonso Martínez-Cruz

**Affiliations:** 1 Liver Disease Laboratory, Center for Cooperative Research in Biosciences (CIC bioGUNE), Bizkaia Science and Technology Park Bld 801A, 48160 Derio, Spain; pgimenez@cicbiogune.es (P.G.-M.); ioyenarte@cicbiogune.es (I.O.); irecio@cicbiogune.es (I.G.-R.); cfernandez@cicbiogune.es (C.F.-R.); mangielitos@hotmail.com (M.Á.C.-R.); igonecamposzarraga@gmail.com (I.C.-Z.); jsimon@cicbiogune.es (J.S.); mlmartinez@cicbiogune.es (M.L.M.-C.); 2 Department of Biochemistry and Goodman Cancer Research Centre, McGill University, Montreal, QC H3A 1A3, Canada; elie.kostantin@mail.mcgill.ca (E.K.); serge.hardy@mcgill.ca (S.H.); michel.tremblay@mcgill.ca (M.L.T.); 3 Instituto de Investigaciones Químicas (IIQ), Centro de Investigaciones Científicas Isla de la Cartuja (cicCartuja), Universidad de Sevilla—CSIC. Avda. Americo Vespucio 49, 41092 Sevilla, Spain; qzaida@us.es (A.D.Q.); idiazmoreno@us.es (I.D.M.); 4 Structural Biology Unit, Center for Cooperative Research in Biosciences (CIC bioGUNE), Bizkaia Science and Technology Park Bld 800, 48160 Derio, Spain; zubimara@hotmail.com (M.Z.L.); nmerino@cicbiogune.es (N.M.); tdierks@cicbiogune.es (T.D.); fblanco@cicbiogune.es (F.J.B.); 5 IKERBASQUE, Basque Foundation for Science, María Díaz de Haro 3, 48013 Bilbao, Spain; 6 Centro de Investigación Biomédica en Red de Enfermedades Hepáticas y Digestivas (CIBERehd), 48160 Derio, Spain; 7 Department of Pediatric Gastroenterology, Nephrology and Metabolic Disorders, Charité Universitäts medizin, 13353 Berlin, Germany; dominik.mueller@charite.de; 8 Istituto di Cristallografia, Consiglio Nazionale delle Ricerche (CNR), Via G. Amendola 122/O, 70126 Bari, Italy; dritan.siliqi@ic.cnr.it

**Keywords:** CNNM4, magnesium, transporter, CNBHD, CBS domain

## Abstract

The four member family of “Cyclin and Cystathionine β-synthase (CBS) domain divalent metal cation transport mediators”, CNNMs, are the least-studied mammalian magnesium transport mediators. CNNM4 is abundant in the brain and the intestinal tract, and its abnormal activity causes Jalili Syndrome. Recent findings show that suppression of CNNM4 in mice promotes malignant progression of intestinal polyps and is linked to infertility. The association of CNNM4 with phosphatases of the regenerating liver, PRLs, abrogates its Mg^2+^-efflux capacity, thus resulting in an increased intracellular Mg^2+^ concentration that favors tumor growth. Here we present the crystal structures of the two independent intracellular domains of human CNNM4, i.e., the Bateman module and the cyclic nucleotide binding-like domain (cNMP). We also derive a model structure for the full intracellular region in the absence and presence of MgATP and the oncogenic interacting partner, PRL-1. We find that only the Bateman module interacts with ATP and Mg^2+^, at non-overlapping sites facilitating their positive cooperativity. Furthermore, both domains dimerize autonomously, where the cNMP domain dimer forms a rigid cleft to restrict the Mg^2+^ induced sliding of the inserting CBS1 motives of the Bateman module, from a twisted to a flat disk shaped dimer.

## 1. Introduction

The Cyclin and CBS domain divalent metal cation transport mediators (CNNMs) are the most recently identified proteins involved in magnesium cation (Mg^2+^) transport across the cell membranes [1,2,3]. The four known members (CNNM1 to CNNM4) encoded in the human genome are integral membrane proteins with strong homology to CorC, a bacterial magnesium/cobalt efflux protein [4]. Despite a remarkable sequence similarity and domain architecture, the CNNMs show an expression pattern that varies significantly in different organs (https://www.proteinatlas.org/search/CNNM) [2,5,6]. For example, CNNM1 is mostly present in the brain, while CNNM2 has also been detected in kidney and liver [4]. In turn, the ubiquitous CNNM3 is additionally expressed in the heart, but scarcely in the skeletal muscle [4], while CNNM4 is predominant in the brain, bone marrow, immune system, and especially abundant in the intestinal tract [4,7]. Since their discovery [4,8], the actual function of CNNM proteins has remained controversial [9,10,11,12,13]. For example, CNNM1 appears to act as a cytosolic copper chaperone [14], while CNNM2 and CNNM4 are commonly considered as basolateral Mg^2+^ extruders (likely Na^+^/Mg^2+^ exchangers) in the renal and intestinal epithelia, respectively [3,15]. Some authors have alternatively proposed them as Mg^2+^ sensors [2] or homeostatic factors that regulate the activity of other still unknown Mg^2+^ transporters [9,16]. Yet, their key role in maintaining Mg^2+^ homeostasis and their involvement in the development of Mg^2+^-related pathologies are undoubted. For example, mutations in CNNM2 or CNNM4 cause recessively inherited dominant hypomagnesemia and renal Mg^2+^ wasting [2] or Jalili Syndrome, respectively [17,18,19,20,21,22,23]. Other disorders related to their altered activity include infertility [24,25], impaired brain development [26] along with neuropsychiatric disorders [27,28,29,30], and abnormal blood pressure levels [31]. On the other hand, CNNM3 and CNNM4 are involved in cancer progression [15,32] by associating with the highly oncogenic phosphatases of the regenerating liver (PRLs) and by promoting intracellular Mg^2+^ accumulation that favors tumor growth and metastasis [6,32,33,34].

**Figure 1:**
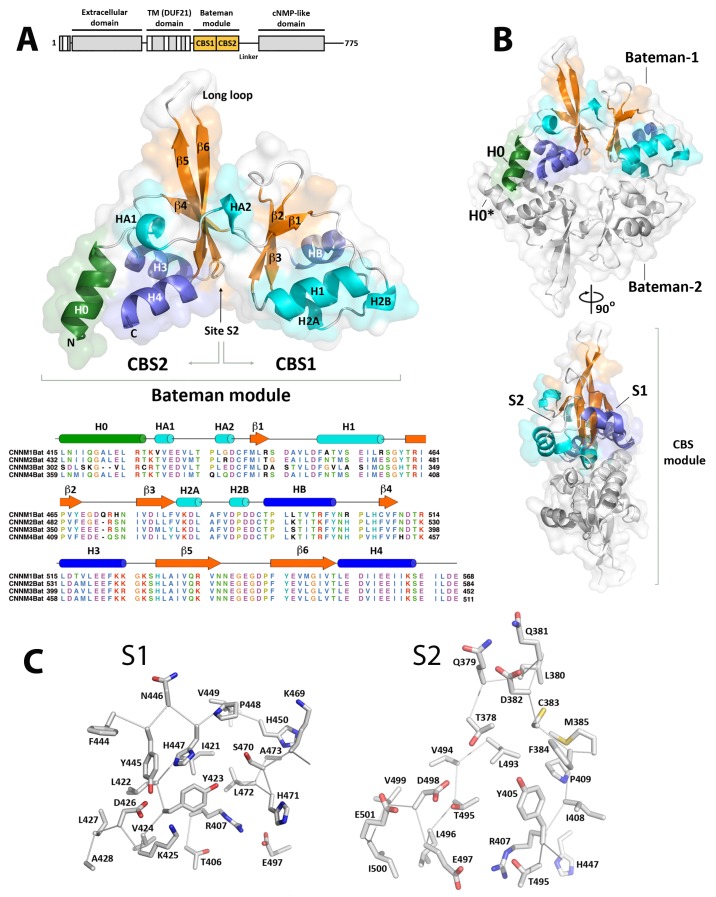
Crystal structure of the hCNNM4 Bateman module. (**A**) (up) Domain architecture of the full length CNNM4 protein (the position of the Bateman module is highlighted in orange). (middle) CNNM4_BAT_ adopts the overall fold of a Bateman module consisting of two consecutive CBS motifs (CBS1, CBS2). The long loop connecting strands β5 and β6 is disordered in most of the crystals. Of note, in CNNM2 and CNNM3 this loop mediates the interaction with all members of the PRL phosphatases family [6,32,33,34,36]. The N-terminal helix H0 connects the Bateman module to the preceding DUF21 transmembrane region in the full-length protein. (down) Sequence alignment of the Bateman modules from human CNNMs. The secondary structure elements (derived from the CNNM4_BAT_ crystal structure) are shown above. (**B**) Two CNNM4_BAT_ Bateman modules (colored vs. grey) associate into a disk shaped homodimer called CBS module. Each CNNM4_BAT_ subunit presents two main clefts on opposite sides of the module (S1 and S2), of which only S2 can host a nucleotide [5,6]. (**C**) Residues forming the S1 and S2 clefts. CNNM4 residue T495 in S2, occupies a position equivalent to that of T568 in CNNM2, where mutation T568I causes hypomagnesemia [2].

Structurally, the CNNMs are complex multidomain proteins that consist of four independent domains connected by linkers of different length (Figure 1). The N-terminal region includes one transmembrane α-helix and an extracellular β-sheet domain with one glycosylation site [2]. A subsequent membrane spanning DUF21 domain (Pfam code PF01595) includes three or four transmembrane α-helices and presumably facilitates Mg^2+^ transport across the cell membrane [2]. The intracellular region is directly attached by a long α-helix (H0) [5] and comprises two distinct domains, a Bateman module and a cyclic nucleotide monophosphate binding-like domain (cNMP domain). The Bateman module consists of two intertwined cystathionine β-synthase (CBS) motifs (CBS1 and CBS2) and binds ATP and Mg^2+^ ions. This triggers important conformational changes that are transmitted to the preceding transmembrane region via the connecting H0 helix and are thought to regulate the transport activity of CNNMs [5,6,35]. The second CBS2 motif features a unique extended loop with a conserved aspartate at its tip. In CNNM2 and CNNM3, this aspartate is solvent exposed and provides the key docking site for stable complex formation with phosphatases of regenerating liver (PRL). Formation of the CNNM2·PRL-1 complex involves structural modifications in both proteins: in PRL-1, the loops defining the entrance to its catalytic cavity shift to interact with the conserved aspartate at the tip of the CNNM2 loop, resulting in a closure of the cavity with inhibition of the phosphatase activity [6,33,34,36]. In CNNM2, electrostatic attraction between surface residues in both proteins causes a displacement of its complementary CBS1 motifs, resulting in a flattening of the complete CBS module [6]. This disk flattening is known to also occur upon MgATP binding that is likely favored by the interaction with the phosphatase [6]. Finally, the C-terminal cNMP domain, also known as cyclic nucleotide binding homology domain (CNBDH), is similar to that present in ion channels and cNMP-dependent kinases [37], and is followed by a long and likely unstructured C-terminal tail (from now on designated as C-tail) with unknown function.

To increase our understanding of the molecular mechanisms underlying CNNM mediated Mg^2+^ transport, analyzed structure and ligand binding of the intracellular region of human CNNM4 (UniProtKB/Swiss-Prot code Q6P4Q7) both in the presence and absence of its known interaction partner, PRL-1 [15]. Thus, we significantly extend the scarce structural data available, which may help in designing new antitumoral drugs to modulate the activity of CNNM/PRL complexes in specific organs.

## 2. Results

A careful analysis of the human CNNM4 amino acid sequence using bioinformatic tools (e.g., Phyre2 [38] and Psipred [39]) predicted the presence of large unstructured stretches in the intracellular region that might impede the crystallization process: (i) a linker connecting the two intracellular domains (residues 512–544), (ii) the C-terminal segment following the cNMP domain (residues 731–775), (iii) a long loop linking the last two β-strands of the CBS2 motif in the Bateman module (residues 480–486), and (iv) an internal loop (residues 639–699) within the core of the cNMP domain. Based on these predictions, we opted for a “divide and conquer” strategy to facilitate crystallization using the following protein constructs: (i) CNNM4_BAT_ (residues 359–511) including the Bateman module and the preceding α-helix H0 connecting it to the DUF21 transmembrane domain in the full-length protein; (ii) CNNM4_cNMP-Ctail_ (residues 545–775) containing the cNMP domain and subsequent C-terminal tail (residues 731–775); (iii) CNNM4_cNMP_ (residues 545–730) containing only the cNMP domain. In addition, we designed constructs comprising both Bateman module and cNMP domain: (iv) CNNM4_BAT-cNMP-Ctail_ (residues 356–775); (v) CNNM4_BAT-cNMP_ (residues 356–730). Of note, we maintained the long loop within the cNMP domain core due to its unknown structural and functional role.

### 2.1. Crystal Structure of CNNM4_BAT_

Crystals of CNNM4_BAT_ were grown as described previously [40]. All crystallographic parameters and refinement statistics are listed in Table 1. The CNNM4_BAT_ domain is formed by two consecutive CBS motifs, CBS1 (residues 371–435) and CBS2 (436–505), that present a αβαββα fold and contact each other via their three-stranded β sheets, resulting in an overall pseudo-C_2_ symmetric structure (upper left panel, Figure 1). As also found in the closest homolog, CNNM2, the strands in the β-sheet of CBS1 are shorter, the N-terminal helix HA1 adopts a stretched 3_10_ conformation, and another 3_10_ helix (H2) following strand β3 is split in two (H2A, H2B). The N-terminal connecting helix H0 packs against helices H3 and H4 of CBS2. Various polar side chains stabilize the Bateman module structure by forming either hydrogen bonds or salt bridges. The former occur more commonly within CBS motifs (i.e., D393-T396 at the N-terminus of helix H1; S388-E412 connecting strands β1 and β2; T495-D498 in the β5-H4 loop) than between CBS motifs (i.e., D426-Y445 connecting helices H2 and HB’, and N360-E463 tying the N-terminal helix H0 to H3). As in other CBS domain containing proteins [41,42], CNNM4_BAT_ features two major symmetry-related clefts, S1 and S2, formed between their stacked β-sheets (central right panel, Figure 1). These clefts contain several basic residues to accommodate phosphonucleotides (Figure 1), although the likely location for their binding in site S1 comprises mainly bulky amino acids (Y423, L472, R407), possibly to prevent the binding of other small molecules here (lower left panel, Figure 1). This steric hindrance is not observed in cleft S2 (lower right panel, Figure 1) that features a hydrophobic environment for adenine binding (F384, M385, Y405, L493, V494) as well as a conserved aspartate (D498) and threonine (T495) for interaction with the ribose moiety. Of note, an acidic cluster (E497, D498, and E501) at the N-terminus of α-helix H4 in S2 replaces the positively charged residues found in S1 (R407, K425, H471, T406). A similar acidic cluster appears to function as a selective filter favoring MgATP over ATP binding in the closest homolog, CNNM2 [5,6]. There, an ATP bound Mg^2+^ cation reduces the electrostatic repulsion between its polyphosphate chain and the acidic cluster. Interestingly, this acidic cluster not only appears in the CNNMs but also in other CBS-containing proteins related with Mg^2+^ extrusion such as the Mg^2+^ tolerance factor SA0657 found in *Staphylococcus aureus* [43], or bacterial proteins CorB and CorC which have been proposed to mediate Mg^2+^ extrusion through bacterial Mg^2+^ channel CorA [44] (Supplementary Figure S1). In other CBS-containing proteins such as the bacterial Mg^2+^ channel MgtE [45,46] or the chloride channels CLCs [47], this acidic cluster is substituted by positively charged residues, which favor the interaction with the triphosphate chain of ATP.

As observed in other CBS-domain containing proteins, including CNNM2 [5,6] and CNNM3 [33], CNNM4_BAT_ self-associates into a disk shaped head-to-head dimer commonly referred to as “CBS module” (upper and central right panels, Figure 1) where both CBS1 and CBS2 pack against their equivalent in the complementary subunit. A closer look at the CBS module in CNNM4_BAT_ reveals some differences from the most common arrangement found in other proteins, in which both complementary Bateman modules lie within the same plane to form a “flat” disk. In CNNM4, this disk is semi-twisted and both CBS1 motifs are slightly separated (Supplementary Figure S2). This particular CBS module represents an intermediate state between the *apo* form of CNNM2 [5], where the separation between complementary CBS1 motifs is more pronounced (“twisted” conformation) [5], and its MgATP bound *holo* form, where the disk appears planar (“flat” conformation) (Supplementary Figure S2) [5]. In the semi-twisted conformation, the CNNM4_BAT_ dimer interface is stabilized by an extensive network of hydrophobic interactions. The contacts between complementary CBS1 motifs are asymmetric, reflecting the intrinsically lower structural order and disruption of helix H2. A hydrophobic cage formed by side chains in helices H1 (F394, M397, M401) and H2 (V424, K425, L427, V430, P432) accommodates residues (A428, F429) in helix H2* of the complementary CBS1* motif. Contrarily, the interaction between both CBS2 motifs is largely symmetric, with reciprocal H3-H4*/H4-H3* helix contacts involving residues L462 in H3 and L496, I500, I503, I504 in helix H4. Upon dimerization, the H0 helices from complementary CNNM4_BAT_ subunits lie crossed (upper right panel, Figure 1), as observed in the twisted conformation of CNNM2_BAT_ [5], and participate in the CBS2 dimer interface via their residues N360, M361, I362, A365, and L368.

### 2.2. CNNM4_BAT_ Interaction with ATP Depends on Mg^2+^

To clarify whether CNNM4_BAT_ shares the Mg^2+^ and/or nucleotide binding ability observed for CNNM2 [5,6,35], we crystallized CNNM4_BAT_ in the presence of ATP and/or different metal ions (Mg^2+^, Zn^2+^, Co^2+^, Ni^2+^), but the obtained crystals had insufficient diffraction quality. We therefore studied Mg^2+^ and ATP binding by solution state NMR spectroscopy, monitoring changes in the 2D ^15^N-HSQC NMR fingerprint spectrum of CNNM4_BAT_ upon ligand addition.

#### 2.2.1. NMR Titration Studies with Mg^2+^

**Figure 2:**
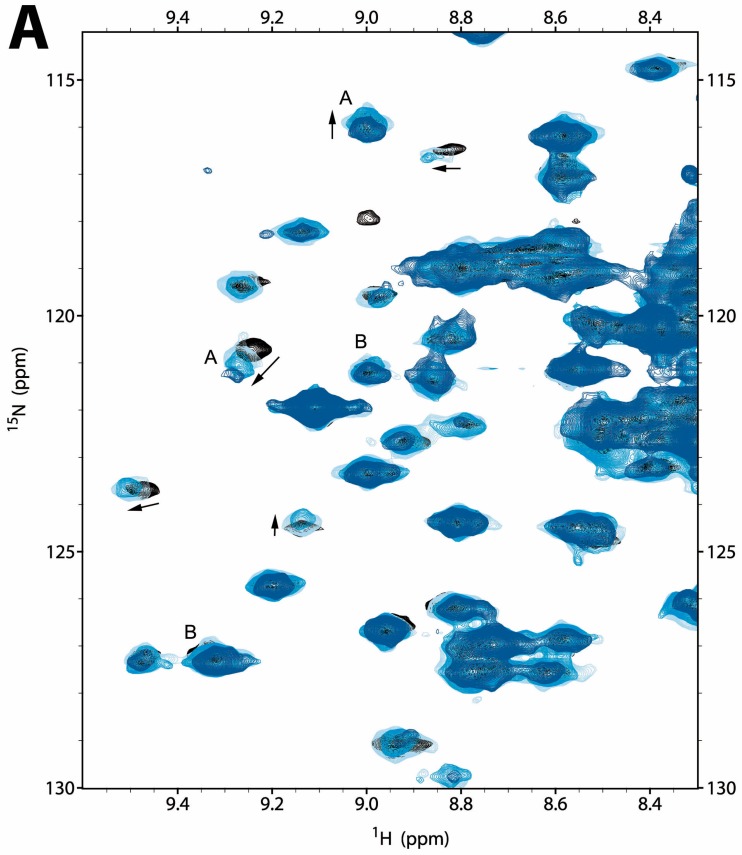

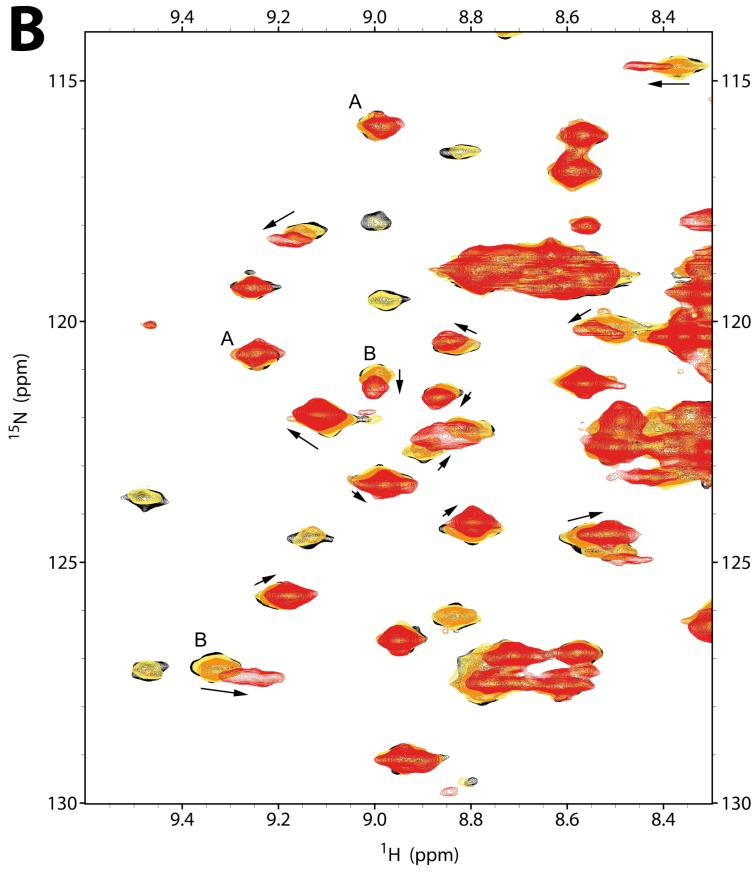

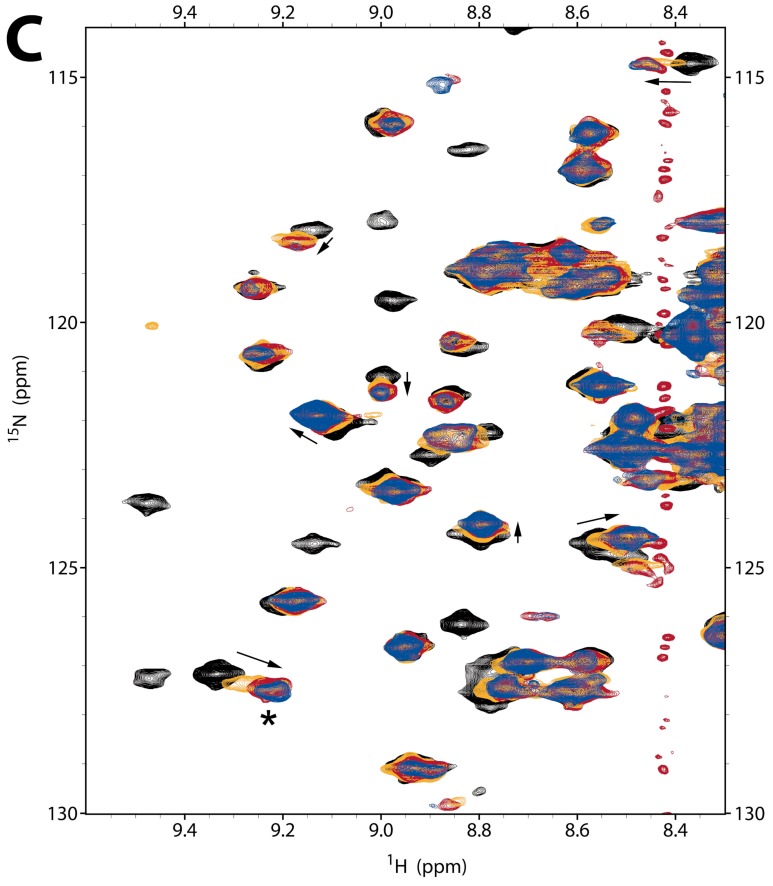
Ligand binding by hCNNM4_BAT_ (100 μM), monitored by 2D ^15^N HSQC NMR spectra (zoom on a representative region). (**A**) Effect of Mg^2+^ addition to a final concentration of 0 mM (black, reference), 10 mM (light blue), 20 mM (blue), and 40 mM (dark blue). Signals shifting or diminishing due to addition of only Mg^2+^ or ADPNP (as in (B)) are marked with letters A or B, respectively, to emphasize differences in the affected signal sets, indicating non-overlapping binding sites. Arrows show the shift direction. (**B**) Effect of ADPNP addition to a final concentration of 0 mM (black, reference), 570 μM (yellow), 1.14 mM (orange), and 5.7 mM (red). (**C**) Effect of combined Mg^2+^ and ADPNP addition: Mg^2+^ plus 0 mM ADPNP (black, reference), 0 mM Mg^2+^ plus 5.7 mM ADPNP (orange), 0 mM Mg^2+^ plus 18 mM ADPNP (red), and 10 mM Mg^2+^ plus 5.7 mM ADPNP (blue). Moderate concentrations of ADPNP (5.7 mM) in the presence of Mg^2+^ produce an effect similar to the addition of high concentrations of ADPNP alone (18 mM; a representative signal is marked by an asterisk). The pertaining full spectra are shown in Figure S2A–C. Spurious t_1_ noise at ^1^H frequencies of ca. 8.2 and 8.4 ppm derive from protons H2 and H8 in the purine ring of ADPNP (see Figure S2D).

The 2D ^15^N-HSQC spectrum of CNNM4_BAT_ (0.1 mM) changed only marginally up to 10 mM MgCl_2_ added, but more substantially at higher stoichiometry (40 mM MgCl_2_) (Figure 2A and Figure S3A). Commonly, relative signal intensities decreased with increasing Mg^2+^ concentration, leading to strong attenuation or even complete disappearance of some signals at 40 mM MgCl_2_. As previously observed for CNNM2_BAT_ [5], the signal attenuation was due to line broadening and/or multiple signal splitting, attesting to slow exchange between free and Mg^2+^ bound forms with significant conformational heterogeneity. Gradual shifting of some signals was also observed (Figure 2A). Overall, our NMR titration studies indicate that CNNM4_BAT_ interacts very weakly with free Mg^2+^ ions, with a K_D_ > 1 mM, on the order of the intracellular Mg^2+^ concentration.

#### 2.2.2. NMR Titration Studies with ADPNP

Similar NMR titration experiments with ATP were compromised by its gradual hydrolysis that caused a continuous decrease of the pH, resulting in widespread false positive changes in the protein’s 2D ^15^N-HSQC spectra. We therefore used adenosyl-β,γ-imidodiphosphate (ADPNP) as a less hydrolysable analog of ATP and increased the HEPES buffer concentration to 100 mM. The chosen pH 7.2 then remained constant up to 6 mM ADPNP concentration and dropped marginally to 7.0 at 18 mM ADPNP concentration, causing only very small shifts of the pH sensitive HEPES ^1^H NMR signals. Compared to Mg^2+^, ADPNP addition produced more significant changes in the ^15^N HSQC spectrum of CNNM4_BAT_, and at distinctly lower concentrations (Figure 2B and Supplementary Figure S3B). Again, line broadening upon ADPNP addition was more prominent and common than signal shifting, with a maximal extent around 5 mM ADPNP concentration suggesting a K_D_ in the range of the intracellular ATP concentration (1–10 mM) [48]. A substantial number of signals (≥20) also shifted gradually with increasing ADPNP concentration, corroborating the estimated K_D_ (Figure 2B and Figure S3B). As described before for CNNM2 [5], and to further confirm ADPNP binding by CNNM4_BAT_, we also performed ^1^H→^1^H Saturation Transfer Difference (STD) NMR experiments that monitor direct polarization transfer from the protein to nearby protons of a reversibly binding ligand. The observed STD effects unambiguously confirmed specific ADPNP binding by CNNM4_BAT_ despite the rather low affinity (Supplementary Figure S2D). Overall, ADPNP binding by CNNM4_BAT_ is rather weak (estimated K_D_ = 10^−2^ to 10^−3^ M), but clearly specific and localized, affecting approximately 15% of all resolved ^15^N HSQC signals (Figure 2B and Figure S3B). The commonly observed line broadening upon ADPNP addition derives from slow exchange between free and multiple bound forms. Of special note, most of the signals affected by ADPNP addition were distinct from those affected by Mg^2+^ addition (compare Figure 2A,B), suggesting different binding sites.

#### 2.2.3. NMR Co-Titration Studies with Mg^2+^ and ADPNP

Adding Mg^2+^ to a mixture of CNNM4_BAT_ and ADPNP (5.7 mM) produced only small further changes in the ^15^N HSQC fingerprint spectrum of CNNM4_BAT_ that affected some 10–15 (i.e., 5%–8%) resolved signals (Figure 2C and Figure S3C). As with Mg^2+^ alone, signal widths and intensities were primarily affected, to a lesser extent also signal frequencies. Interestingly, the small spectral changes from Mg^2+^ co-addition made the ^15^N HSQC spectrum closely resemble the one obtained at very high ADPNP concentrations (18 mM). Thus, Mg^2+^ co-addition enhances and stabilizes the spectral effects of ADPNP binding to CNNM4_BAT_, suggesting a positive cooperative effect of Mg^2+^. Presumably, the increased conformational heterogeneity of CNNM4_BAT_ induced by Mg^2+^ addition facilitates a conformational selection mechanism for ADPNP binding. Since binding of ADPNP and Mg^2+^ largely affect different sets of CNNM4_BAT_ signals, their binding sites do not seem to overlap (Figure 2A–C). Based on the crystal structures of CNNM4_BAT_ (presented here) and CNNM2_BAT_ [5,6], we conclude that an elevated Mg^2+^ concentration neutralizes the charge repulsion between nucleoside phosphate groups and acidic residues at site S2 of CNNM4_BAT_, and contributes to stabilize the “flat” conformation of the CBS module.

### 2.3. Crystal Structure of CNNM4_cNMP_

**Figure 3:**
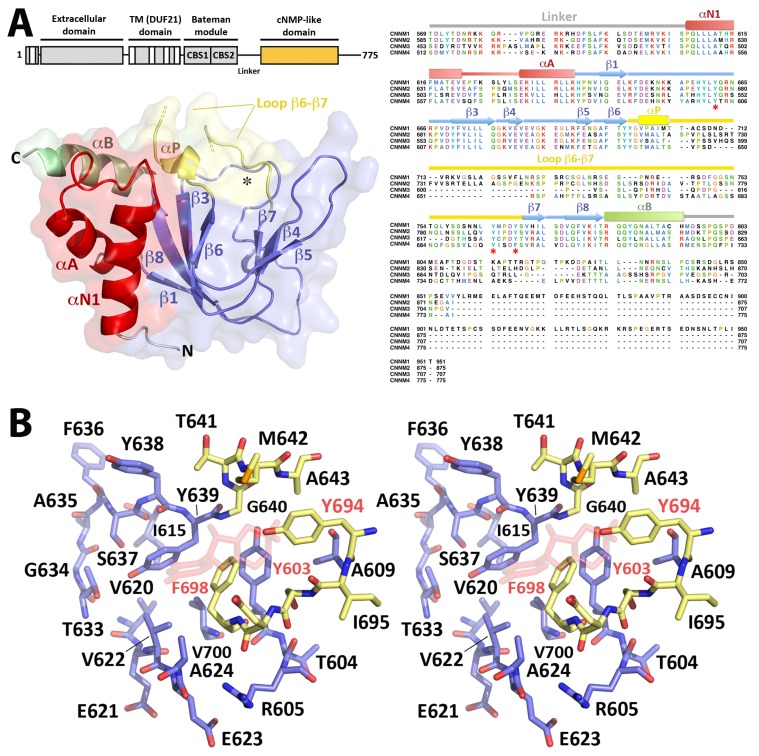
Crystal structure of the *h*CNNM4_cNMP_ domain. (**A**) (left) The hCNNM4_cNMP_ crystal structure shows two N-terminal helices (αN1, αA; in red), followed by a central seven-stranded β-roll (blue) and a C-terminal helix αB (green). The β-roll shows a cavity (marked by an asterisk) presenting the so-called phosphate binding cassette (PBC, in yellow) formed by a short helix (αP) and an extended loop (ca. 60 residues, invisible in the crystals) between strands β6 and β7. The domain architecture of the full length protein is shown above (the cNMP domain is highlighted in orange). (Right) Sequence alignment of cNMP domains in human CNNMs. The long β6-β7 loop is conserved throughout, but is larger in CNNM1 and CNNM2, intermediate in CNNM4, and shorter in CNNM3. A likely unstructured polypeptide segment following helix αB was not included in our CNNM4_cNMP_ construct. The colors of the secondary elements correspond to those used in the crystal structure. (**B**) Stereo view of residues at the cavity opening of the β-roll in CNNM4_cNMP_. Residues within β-strands or the PBC motif are shown in blue or yellow, respectively. Residues Y603, F698, and Y694 (labeled in red and indicated by a red asterisk in the sequence alignment) invade the space usually occupied by a bound cyclic nucleotide in unrelated CNB containing proteins like PKA (a superimposed cAMP molecule is shown by transparent red sticks).

Crystallization of the cNMP binding domain of CNNM4 was only possible using a CNNM4_cNMP_ construct without the C-terminal tail. This segment is the most variable region in all known cyclic nucleotide binding domains (CNBD) and, in other proteins, usually contains a αC helix that helps to position a bound cyclic nucleotide. A secondary structure analysis predicted the C-tail to be unstructured in all CNNMs, thus explaining our failed crystallization attempts with the larger CNNM4_cNMP-Ctail_ construct. Our final CNNM4_cNMP_ model was refined to 3.7 Å resolution (Table 1 and Figure 3) and shows an overall fold similar to the CNBD domains in, e.g., PKA, cAMP activated exchange proteins, cyclic nucleotide gated (CNG) as well as hyperpolarization activated cyclic nucleotide gated (HCN) channels, and also in proteins containing CNBD homology domains (CNBDH) such as the KCNH voltage-dependent potassium channels [49,50,51]. As in these proteins, CNNM4_cNMP_ features a central β-roll surrounded by helical elements at both its N- and the C-terminus. The two N-terminal helices αN1 and αA, preceding strand β1, run antiparallel (Figure 3). Helix αA furthermore packs antiparallel against the C-terminal helix αB that immediately follows strand β8. The β-roll contains the so-called phosphate binding cassette (PBC) [49] formed by strand β6, a helix turn (αP), and strand β7 (Figure 3). In CNNM4_cNMP_, a network of H-bonds between Y603 (β1), Y611 (β3), and Y639 (αP) as well as between Y639 (αP) and L602 (β1) stabilizes this motif and defines the separation between β-sheets, thus, the size of the main cavity. Another H-bond network formed by residues T633 (β5), G634, S637 (β6), and Y639 (αP) stabilizes a tight turn of the loop connecting strands β5 and β6.

#### 2.3.1. The CNNM4_cNMP_ Module Is Unable to Bind Cyclic Nucleotides

CNNM4_cNMP_ has unique features that distinguish it from canonical CNBD and CNBDH domains. The first striking difference is the unusual length of the loop connecting strands β6 and β7 in the PBC, which is approximately 50 amino acid residues longer than in related proteins. This anomalously long loop, disordered and not visible in our crystal structure, is conserved in the four members of human CNNM family. The second unique feature in CNNM4_cNMP_ is an abrupt turn of the polypeptide chain at residues 601–603 that distorts the otherwise canonical strand β2 and redirects the Y603 side chain towards the interior of the cavity, thus blocking the required space to accommodate a cyclic nucleotide like cAMP or cGMP (Figure 3). Two further structural details corroborate our assumption that the cNMP binding domain in CNNM4 (and, likely, in all CNNMs) neither binds nor is regulated by cyclic nucleotides: first, CNNM4_cNMP_ does not contain a conserved buried arginine (e.g., R301 in PKG1, Supplementary Figure S4) to interact with the exocyclic phosphate of cAMP or cGMP, nor the glutamate to fix the orientation of their ribose 2”-OH (E292 in PKG1, Supplementary Figure S4). These residues are substituted by Ser/Pro and Thr/Val, respectively, in the human CNNMs (Figure 3 and Figure S4). Second, the base of the β-roll in CNNM4_cNMP_ is occupied by a network of bulky residues (Y603, Y694, F698) that form a hydrophobic pocket along with residues L602, I613, V620, V622, and V700 (Figure 3 and Figure S4), which sterically hinders nucleoside binding inside the cavity. Analogous bulky residues are present in all known CNNM homologs, even from phylogenetically distant organisms (Supplementary Figure S5). To confirm the suggested inability of CNNM4_cNMP_ to bind nucleotides we performed isothermal titration calorimetry (ITC) and titration studies by NMR. The thermograms and isotherms of cAMP or cGMP titration to CNNM4_BAT-cNMP-Ctail_ (Supplementary Figure S6) show that the injection heat remains constant and low (as for simple water injection into water), indicating the absence of specific nucleotide-protein interactions under our experimental conditions. The absence of any saturation behavior in the isotherms made model fitting unnecessary. In agreement with these results, no significant spectral changes were observed in the 2D ^15^N HSQC spectra of CNNM4_cNMP-Ctail_ upon addition of cAMP or cGMP (data not shown). Our results are in agreement with thermal shift assays (TSA) carried out by Chen et al., demonstrating the lack of cyclic nucleotide binding in the cNMP domains of CNNM2 and CNNM3 [52].

#### 2.3.2. CNNM4_cNMP_ Forms Symmetric Homodimers

**Figure 4:**
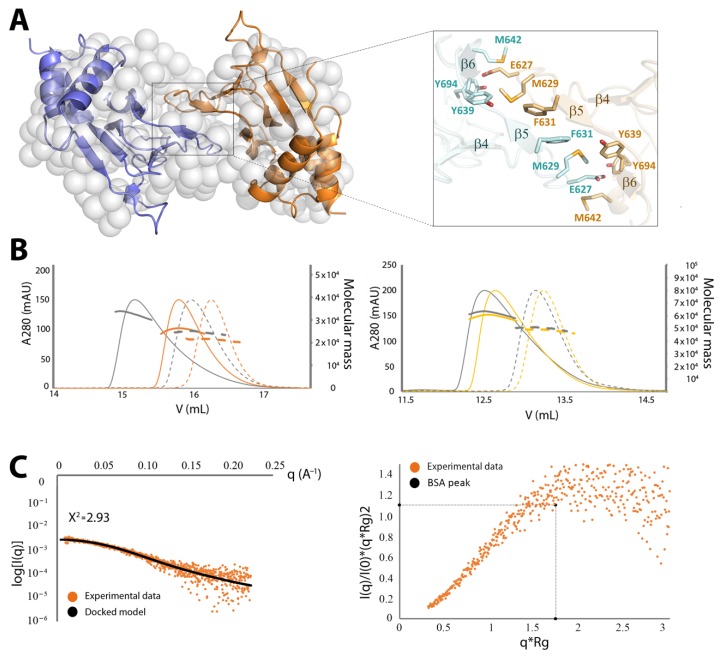
Structure of the CNNM4_cNMP_ dimer. (**A**) (left) Association of two CNNM4_cNMP_ domains (blue and orange) in the crystal. The presence of such dimers in solution was confirmed by SAXS data that indicate a shape and volume consistent with the shown bead model (grey spheres). (**A**) (right) Residues (mostly hydrophobic) at the dimerization interface between CNNM4_cNMP_ subunits, formed primarily by their strands β4 and β5. (**B**) SEC-MALS analysis of CNNM4_cNMP_ (left) and CNNM4_BAT-cNMP-Ctail_ (right). CNNM4_cNMP_ (grey; Mw = 20936.78 Da monomer) yields an apparent Mw of 24,740 ± 60 Da and 33,920 ± 50 Da at concentrations of 1 mg/mL (dotted lines) and 10 mg/mL (solid lines), respectively, indicating prevalence of the dimer at higher concentration. For the F631A mutant (orange), smaller apparent M_W_ of 21,730 ± 50 Da and 26,440 ± 50 Da are observed for 1 and 10 mg/mL, respectively, indicating that this mutation strongly impedes dimerization. CNNM4_BAT-cNMP-Ctail_ (grey; M_W_ = 47,861.17 Da monomer) similarly yields an apparent Mw of 51,120 ± 60 Da and 63,610 ± 100 Da for 1 and 10 mg/mL, respectively, again indicating prevalence of the dimer at higher concentration. For its F631A mutant (yellow), very similar M_W_ of 49,230 ± 80 Da and 60,920 ± 90 Da are derived, indicating a negligible effect on CNNM4_BAT-cNMP-Ctail_ dimerization. Thus, the disruption of CNNM4_cNMP_ dimerization by this mutation (see left) is overridden by dimerization via the still intact Bateman module. (**C**) SAXS analysis of CNNM4_cNMP_. (left) Experimental SAXS data (orange) and scatter curve (black) for the best-fit bead model (Χ^2^ = 2.93) of CNNM4_cNMP_ dimers in solution (shown in (**A**)). (right) The dimensionless Kratky plot (orange) indicates a well-folded, flexible, and elongated arrangement for CNNM4_cNMP_. The corresponding BSA reference peak is also shown.

A SEC-MALS analysis revealed that isolated CNNM4_cNMP_ in solution exists as a concentration dependent mixture of monomers and dimers (Figure 4). Dimer formation was confirmed by small angle X-ray scattering (SAXS) (Figure 4, Table 2), which allows to assess macromolecular flexibility, shape, and assembly at low resolution [53]. The dimensionless Kratky plots and Porod exponent (2.3) revealed a well-folded, flexible, and elongated arrangement for CNNM4_cNMP_ (Figure 4, Table 2). An analysis of all interfacing molecules in the CNNM4_cNMP_ crystal structure also suggested homodimeric assemblies, where the intersubunit interface of 617 Å^2^ is mostly hydrophobic and involves residues E627, M629, F631, Y639, M642, and Y694 (Figure 4). M629 (in the loop preceding strand β5) points towards the hydrophobic pocket formed by F631 (β5), E627 (loop β4-β5) and, in the complementary subunit, V622, Y638 (β4), Y639 (loop β6-αP) (Figure 4). A network of H-bonds involving residues E627 (loop β4-β5), T633, G634, S637, Y639, Y694 (loop β6-β7), and K626 (loop β4-β5) as well as an additional salt bridge between K626 and D697 (loop β6-β7) contribute to stabilize the dimer. Interestingly, mutating the interface residue F631 to alanine yielded mostly monomers, highlighting its relevant role in stabilizing the dimer via π-stacking with its counterpart. A main feature in the dimer is the presence of two large symmetrical cavities of approximately 825 Å^3^, separated by complementary strands β4 and β5, whose walls are formed by both CNNM4_cNMP_ subunits via their strands β4, β5, the loop following helix αP, and the N-terminal helix αN1 (Figure 4 and Supplementary movie S1).

Since the CNNM4_BAT_ domain likewise dimerizes, we further investigated which of the two intracellular modules (Bateman or cNMP) governs the dimerization of the complete intracellular region. For this, we evaluated the impact of the aforementioned F631A mutation (in the cNMP domain) in the larger construct CNNM4_BAT-cNMP-Ctail_ that, like CNNM4_cNMP_, also shows a concentration dependent mixture of monomers and dimers (Figure 4). In contrast to the latter, however, CNNM4_BAT-cNMP-Ctail_ is barely affected by F631A (Figure 4) mutation indicating that the Bateman module (CNNM4_BAT_), rather than the cNMP domain (CNNM4_cNMP_), plays the more important role in CNNM4 dimer stabilization.

### 2.4. Structural Model of the Complete Intracellular Region of CNNM4

**Figure 5:**
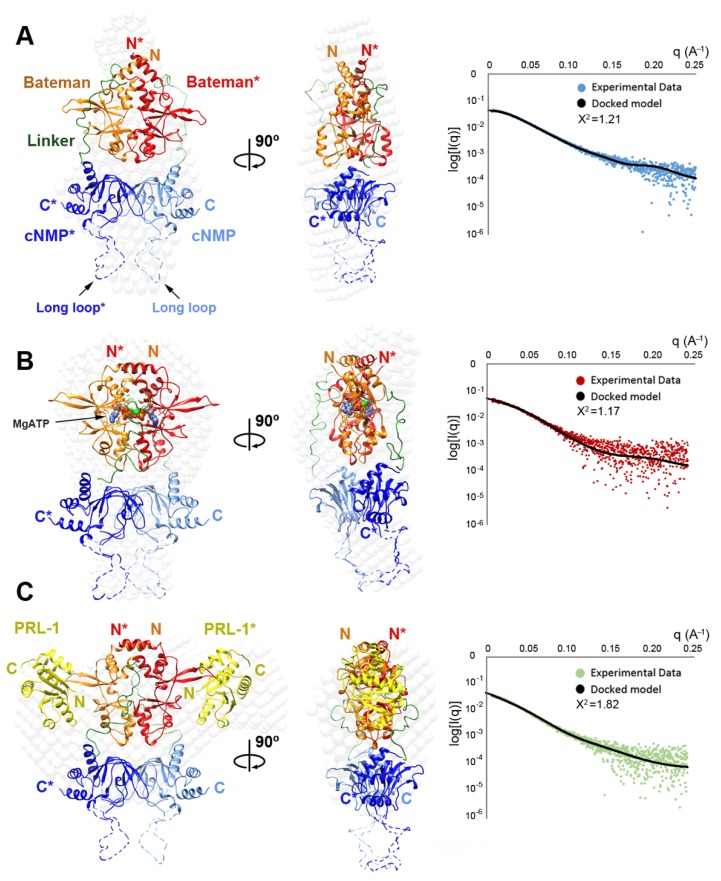
Solution structure of CNNM4_BAT-cNMP-Ctail_ alone and in complex with PRL-1. (**A**) SAXS derived bead model of CNNM4_BAT-cNMP-Ctail_ in the absence of MgATP, built from the crystal structures of the isolated CNNM4_cNMP_ (PDB ID 6G52) and CNNM4_BAT_ in twisted conformation (modeled from CNNM2_BAT_, PDB code 4IYS). (**B**) SAXS derived bead model of CNNM4_BAT-cNMP-Ctail_ in the presence of MgATP, built from the independent crystal structures of the isolated CNNM4_cNMP_ and CNNM4_BAT_ in flat conformation (modeled from CNNM2_BAT_, PDB code 4P1O). The invisible disordered C-terminal tail of CNNM4_cNMP_ is not represented for clarity. (**C**) SAXS derived bead model of the complex formed by PRL-1 and CNNM4_BAT-cNMP-Ctail_ in the presence of MgATP. The model was built from the crystal structures of the homologous CNNM2_BAT_ complexed with both PRL-1 and ZnATP (PDB ID: 5LXQ) and CNNM4_cNMP_ (PDB ID: 6G52). In all structures shown, the disordered linker (green) between Bateman module and cNMP domain was modelled with CORAL [54], the putative position of the long loop within the cNMP domain is represented as a dotted line. The complementary monomer is distinguished by an asterisk. Panels to the right show the corresponding SAXS data and computed scatter curves (black) for the illustrated best-fit bead model.

All our attempts to grow crystals from constructs containing both intracellular modules (i.e., CNNM4_BAT-cNMP_ and CNNM4_BAT-cNMP-Ctail_) failed, presumably due to their monomer/dimer equilibrium in solution and the presence of disordered regions such as the interdomain linker, the long loop connecting strands β6 and β7, and the C-terminal tail following the cNMP binding domain. We therefore proceeded with a structural characterization by SEC-SAXS of the CNNM4_BAT-cNMP-Ctail_ construct in the absence and presence of MgATP. In agreement with the crystallographic data from the isolated domains, the derived R_g_, D_max_, and Mw values (estimated from the Porod volume) are consistent with a dimeric assembly under all experimental conditions (Table 2). The Porod exponent, used to estimate the flexibility of the protein, indicates a rather compact and rigid molecule (Table 2). The dimensionless Kratky plot (Supplementary Figure S7), used to assess the shape of a protein, shows a higher and right-shifted maximum compared to the reference BSA (maximum = 1.104 at √3), thus indicating a well-folded and elongated molecule (Supplementary Figure S7). Using the isolated crystal structures of CNNM4_BAT_ and CNNM4_cNMP_, and the bead model (C-α low resolution model as described in §4.5) obtained from the SAXS experimental data, we next constructed a structural model of the whole intracellular region of CNNM4 (Figure 5). Three possible different scenarios were considered during the construction and refinement of such model: (1) in the first one, both CNNM4_BAT_ and CNNM4_cNMP_ dimers were treated as independent rigid bodies; (2) in the second, only CNNM4_BAT_ dimers (CBS modules) were predefined while the CNNM4_cNMP_ subunits were treated as independent, not necessarily interacting entities; (3) inversely, CNNM4_cNMP_ dimers were predefined while the complementary CNNM4_BAT_ modules were treated as independent, not necessarily interacting entities. The structure coordinates used for the CNNM4_cNMP_ dimer were taken from our PDB entry 6G52. For scenarios (1) and (2), two possible conformations of the CBS module were considered: (i) a twisted disk, as observed in the closest homolog, CNNM2, in the absence of MgATP (PDB ID: 4IYS) [5]; (ii) a flat disk, as observed in the MgATP-CNNM2_BAT_ complex (PDB ID: 4P1O) [5] (note: the MgATP-CNNM4_BAT_ crystal structure remains unknown). We then explored all possible relative orientations of the intracellular domains without including the interdomain linker nor the C-terminal tail.

**Figure 6:**
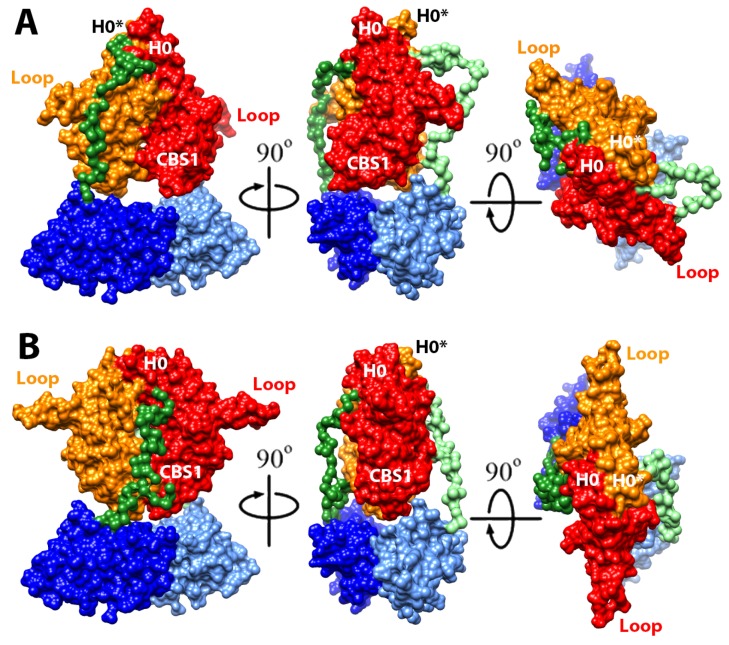
Structure of the complete intracellular region, CNNM4_BAT-cNMP-Ctail_. The picture shows three orthogonal views in the absence (**A**) and presence (**B**) of bound MgATP. The structures were derived from SAXS and high resolution crystallography data. The CBS module, formed by two associated Bateman modules (red and orange), lies above their likewise dimerized CNNM4_cNMP_ binding domains (dark and light blue), to which they are connected by an extended linker (dark and light green). The CBS1 motif in each Bateman module first into the large central cavity formed by the associated CNNM4_cNMP_ domains. This contiguous and rigid cleft is morphologically complementary to both CBS1 motives and restricts their movement during the transition from twisted (as in (A)) to flat (as in (B)) conformations upon MgATP binding (at site S2 between connected CBS1 and CBS2 motives, see Figure 1). Helices H0, located distal from the central cavity, connect the Bateman modules to the preceding DUF21 transmembrane region.

The arrangements that best fit the SAXS data were assemblies with 2-fold symmetry according to scenario (1), in which both CNNM4_BAT_ and CNNM4_cNMP_ associate with their corresponding counterparts. All models with an average Χ^2^ < 2 showed the CBS1 motifs above the large cavity between both CNNM4_cNMP_ domains (Figure 5 and Figure 6). After determining the relative orientation of the intracellular domains, we progressively rotated the CNNM4_cNMP_ dimer around the 2-fold axis relating both Bateman modules until the best fit was obtained. Of two different rotamers, both with Χ^2^ ≈ 1.3 but differing by 60°, one showed larger morphological complementarity between the two intracellular domains and was, therefore, selected as the final model (Figure 5 and Figure 6, Supplementary movies S1 and S2, Table 2). Finally, the relative distance between the dimerized CNNM4_BAT_ and CNNM4_cNMP_ domains along the 2-fold axis was refined, and the polypeptide chain connecting both intracellular domains as well as the C-terminal tail following CNNM4_cNMP_ were modeled using the CORAL program [54].

### 2.5. Structural Model of the Full Intracellular Region of CNNM4 in Complex with PRL-1

CNNMs are known to interact with phosphatases of the PRL family [6,33,34,36] via the extended loop in their CBS2 motif and the catalytic cavity in PRL. Formation of the CNNM·PRL complex involves structural modifications in both proteins: in PRL, the loops defining the entrance to its catalytic cavity shift to interact with a conserved aspartate at the tip of the CNNM loop (D558 in CNNM2), resulting in a closure of the cavity with inhibition of the phosphatase activity [6,33,34,36]. In CNNM, electrostatic attraction between surface residues in both proteins causes a displacement of its complementary CBS1 motifs, resulting in a flattening of the complete CBS module [6]. This disk flattening is known to also occur upon MgATP binding that is likely favored by the interaction with the phosphatase [6]. Based on this knowledge, we therefore analyzed the complex formed by PRL-1 and the CNNM4_BAT-cNMP-Ctail_ construct, both in the absence and in the presence of MgATP. As expected, the obtained R_g_ and D_max_ values were very similar in both cases (48 and 185 Å vs. 47 and 183 Å, respectively). The Porod volume indicated that one CNNM4_BAT-cNMP-Ctail_ dimer bound to two separate PRL-1 molecules (Table 2 and Figure 5) while the dimensionless Kratky plot showed a maximum higher than the BSA standard, consistent with a well-folded and elongated shape (Supplementary Figure S7, Table 2). To then model the CNNM4_BAT-cNMP-Ctail_·PRL-1 complex structure, we started from two building blocks: (i) the dimeric CNNM4_BAT_ domain was replaced by the flat CBS module of the CNNM2∙PRL-1 complex (PDB ID: 5LXQ) [6] after substituting the 14 differing residues; (ii) the CNNM4_cNMP_ dimer was taken from its isolated structure described here (PDB ID: 6G52). Both rigid building blocks were iteratively rotated against each other; the resulting complex structures where then analyzed with ZDOCK [55] and UCSF CHIMERA [56], and refined against the SAXS data. In a first round, all assemblies with Χ^2^ values < 2.5 (relative to the SAXS data) were selected as suitable candidates for subsequent refinement. We then explored all possible rotations between both building blocks in the dimeric assemblies, maintaining the 2-fold symmetry between monomers. The lowest Χ^2^ was obtained for two similar structure models differing by a 60° rotation angle of the CNNM4_cNMP_ dimer around the 2-fold symmetry axis, of which we selected the model with the highest morphological complementarity between both building blocks (Figure 5). In a last step, we used CORAL [54] to model the linkers between the CNNM4_BAT_, CNNM4_cNMP_, and C-terminal tail regions. The resulting final structure model of the CNNM4_BAT-cNMP-Ctail_·PRL-1 complex (Figure 5, Supplementary movie S3) is a pseudo symmetric assembly in which the Bateman modules and cNMP binding domains are related by a 2-fold axis running between the interfacial helices of the Bateman domain. The cNMP binding domains localize close to the CBS1 motifs of the Bateman domains and away from their H0 helices that connect the intracellular region with the transmembrane DUF21 module. As expected [6], the PRL-1 molecules do not interact with the cNMP binding domains that, in turn, do not tightly pack against the Bateman modules (Figure 5, Supplementary movie S3).

## 3. Discussion

The biological relevance of CNNM proteins in cellular magnesium transport as well as their role in diverse pathological processes like magnesium wasting, neurological disorders, and cancer has become evident in recent years. Identification of some of their molecular partners and the metabolic and phenotypic effects resulting from their inhibition has shed some light on functional questions that arose immediately after their discovery. Despite significant experimental efforts, however, the actual role played by these homeostatic factors in different organs and cell types as well as the molecular mechanisms by which they (help to) mediate Mg^2+^ transport across the cell membranes remain a subject of intense debate. In this regard, high resolution structure information would be very helpful, but crystallization of these multimodular transmembrane proteins is greatly impeded by their intrinsically dynamic nature. By exploiting the structural independence of their different domains and applying a “divide and conquer” approach, we managed to obtain a structure model of the complete intracellular region of human CNNM4. The presented results provide broad structural insight on the CNNM family and reveal the relative orientation of their different domains in the absence and presence of PRL-1, an oncogenic phosphatase of regenerating liver that gets inhibited by CNNM binding and, inversely, inhibits their activity in Mg^2+^ transport. Our crystal structures show that the Bateman module of CNNM4 forms two main clefts, S1 and S2, where S2 may accommodate a bound nucleotide despite an unusually acidic surface rarely seen in typical ATP binding sites. The resulting electrostatic repulsion between the cluster of negatively charged residues in S2 and the ATP polyphosphate chain may explain the low nucleotide affinity and its increase by Mg^2+^ co-binding for charge compensation, as shown by our NMR titration experiments. While, in principle, the low ATP affinity of CNNMs is unexpected for Mg^2+^ transporters or sensors, it might be relevant for their sensor function in order to allow transport of Mg^2+^ cations only above a certain intracellular concentration (in the millimolar range). Of relevance in this regard, the Mg^2+^ dependence of ATP binding by CNNMs is exactly inverse to that by bacterial channels, which regulate the intracellular Mg^2+^ concentration by controlling the cation influx, rather than efflux. For instance, bacterial MgtE requires prior ATP binding to the Bateman module in order to enhance its Mg^2+^ affinity. This cooperative binding then promotes a conformational change leading to a closure of the MgtE membrane pore above a certain intracellular Mg^2+^ concentration [46,48].

Furthermore, as observed in most CBS domain containing proteins [41,42], our crystallographic and SEC-MALS data confirm that the Bateman module also plays a key role in CNNM4 dimerization. Remarkably, we found that the CNNM4_BAT_ dimer assembles in a semi-twisted disk conformation resembling an intermediate state between the fully twisted and flat disks adopted by the CNNM2_BAT_ dimer in the absence and presence of MgATP/Mg^2+^, respectively (Supplementary Figure S2) [5]. Considering the high sequence identity between CNNM2 and CNNM4 Bateman modules (where only 14 residues are different) it is reasonable to assume that, in solution, the intermediate state seen in the CNNM4_BAT_ crystals also evolves into the fully twisted conformation in the presence of MgATP/Mg^2+^.

On the other hand, our crystal structure of CNNM4_cNMP_ as well as ITC and NMR titration experiments reveal that this cyclic nucleotide binding-like domain (CNBH) actually cannot accommodate and/or be regulated by cAMP or cGMP, contrary to its name (Supplementary Figure S6). Interestingly, CNNM4_cNMP_ associates in compact homodimers (Figure 4 and Supplementary movie S1) that are equally seen in the dimer structures of CNNM2 and CNNM3 [52]. Thus, and in line with these recent findings on the homologs, CNNM4_cNMP_ appears to contribute importantly to the dimerization of the full-length protein while our SEC-MALS and SAXS data on the larger CNNM4_BAT-cNMP-Ctail_ construct (containing both Bateman and cNMP domains) support a complementary and non-negligible role in dimer stabilization also for the Bateman module. Importantly, our SAXS derived structure model suggests that the CNNM4_cNMP_ domain dimerization defines the morphological limits for the twisted-to-flat conformational change triggered within the Bateman module by MgATP binding in their S2 sites: we posit that the contiguous large cavity formed between both CNNM4_cNMP_ monomers sets the limits for the induced shifting of the CBS1 motifs that insert into it (Figure 6 and Supplementary movies S1–S4). Supporting this important dimer stabilizing and motion restricting function by the cNMP domain, its removal inhibits Mg^2+^ transport by CNNM2 and CNNM3 [52]. We moreover found that mutation F631A (in its strand β5) induces the disassociation of CNNM4_cNMP_ dimers, although dimers of larger constructs including also the Bateman module may still form.

In conclusion, we elucidated the overall architecture of the complete intracellular region of human CNNM4, alone and in complex with the binding partner PRL-1. Our data shows cooperative ATP and Mg^2+^ binding by the Bateman module (in site S2), providing a rationale for the observed Mg^2+^ concentration sensitivity, while the so-called cNMP binding domain is actually unable to bind cyclic nucleotides. Both modules dimerize autonomously, thus contributing to overall dimer stabilization in the full-length protein. Yet, while the Bateman module dimer is conformationally more flexible, the cNMP domain dimer is rigid and, thus, provides a stable framework delimiting the conformational changes induced in the former by MgATP binding. These results may pave new ways for designing drugs aimed at modulating the activity of CNNM4 and, by extension, of its closest family members.

## 4. Materials and Methods

### 4.1. Cloning and Protein Purification

The cDNA of human CNNM4 (hCNNM4) was purchased from OriGene (MD, USA; catalogue No. SC113208). The constructs hCNNM4_BAT-cNMP-Ctail_ (residues 356–775), hCNNM4_cNMP_ (residues 545–730), and hCNNM4_cNMP-Ctail_ (545–775), were cloned into pHIS-Parallel2 [57], confirmed by sequencing, expressed in *Escherichia coli* BL21 (DE3), and grown in Luria–Bertani (LB) broth at 37 °C after induction at OD_600_ = 0.8 by adding 0.5 mM isopropyl-β-D-thiogalactopyranoside (IPTG). Cells containing the target proteins were harvested after 4 h of growth at 37 °C. Seleno-L-methionine (SeMet) derivates of the target proteins were similarly expressed in *E. coli* B834 (DE3) cells that were harvested, resuspended in SeMet medium base plus nutrient mix (Molecular Dimensions), and starved of methionine for 1 h. Then, 0.2 mM SeMet (Acros Organics, NJ, USA) and 0.5 mM IPTG were added to the medium and the cells were again harvested after 4 h of growth at 37 °C. The following purification steps were performed at 4 °C.

Both native and SeMet labeled proteins were purified by the same protocol, with few modifications. The cell pellet was resuspended in lysis buffer (50 mM Tris-HCl, pH 8, 300 mM NaCl, 50 mM imidazole, 1 mM DTT, 1 mM benzamidine, 0.1 mM PMSF) and lysed by sonication in a Labsonic P sonicator (Sartorius; 12 cycles of 15 s at 60% amplitude, with 60 s resting on ice between each cycle to prevent sample overheating). The lysate was clarified by ultracentrifugation at 250,000× *g* for 60 min at 4 °C and the supernatant was filtered through a 0.45 μm filter before injection onto a pre-equilibrated 5 mL HisTrap HP column (GE Healthcare), connected to an AKTA FPLC system (GE Healthcare) inside a cold room at 4 °C. The column was then washed with 10 column volumes of buffer A (50 mM Tris-HCl, pH 8, 300 mM NaCl, 50 mM imidazole, 1 mM DTT) and the bound protein was eluted with buffer B (50 mM Tris-HCl, pH 8, 300 mM NaCl, 300 mM imidazole, 1 mM DTT) using a linear gradient over 30 column volumes. Fractions containing the protein of interest (as confirmed by SDS-PAGE) were pooled and TEV protease was added to remove the N-terminal His-tag. The mixture was dialyzed overnight in 50 mM Tris-HCl pH 8, 150 mM NaCl, 1 mM DTT. A second Ni^2+^-NTA chromatography was carried out to remove cleaved 6xHis-tag and uncleaved protein. The cleaved protein was subsequently concentrated in an Amicon Ultra-15 (10,000 Da cutoff) centrifugal concentrator (Millipore) to a volume of ca. 2 mL, then loaded onto a HiLoad Superdex 75 16/60 Prep Grade gel filtration column (GE Healthcare) pre-equilibrated with 20 mM HEPES pH 7.4, 200 mM NaCl, 1 mM DTT, and eluted at a flow rate of 0.3 mL/min. Fractions containing pure protein were pooled and concentrated to 8–10 mg/mL using an Amicon Ultra-4 5000 Da cutoff concentrator (Millipore), and were flash frozen in liquid nitrogen and stored at −80 °C. The final concentration of all purified proteins was estimated by measuring the corresponding OD_280_ at 280 nm and applying the theoretical extinction coefficient.

Human CNNM4_BAT_ was obtained by previously published protocols [40]. For NMR studies, an alternative construct of hCNNM4_BAT_, containing a N-terminal His6-tag with V5 epitope and Tobacco Etch Virus (TEV) proteinase cleavage site, was cloned in a pET151-D/TOPO vector (Invitrogen). The DNA sequence was then transformed into the *E. coli* strain BL21 CodonPlus (Stratagene) and the overexpression procedure was slightly modified from Marley’s protocol [58]. After growth at 37 °C to an OD_600_ of 0.6 in LB media containing 0.1 mg/mL ampicillin, the culture was concentrated threefold into M9 media with ^15^NH_4_Cl plus 0.2 % glucose to produce ^15^N-labeled His-hCNNM4_BAT_, and equilibrated at 20 °C for 30 min before overnight induction with 0.5 mM IPTG at 20 °C. Cells were then lysed by sonication in 25 mM HEPES pH 7.4, 400 mM NaCl, 20 mM imidazole, 1 μM β-mercaptoethanol, 0.1 mM PMSF, 1 mM benzamidine plus DNAse. After centrifugation at 35,000× *g* for 30 min, the extract was immediately subjected to affinity chromatography on a 1 mL HisTrap FF crude (GE Healthcare) column pre-equilibrated in the same buffer, but without DNAse. The protein was eluted by 25 mM HEPES pH 7.4, 0.4 M NaCl, 500 mM imidazole, 1 μM β-mercaptoethanol, 0.1 mM PMSF, 1 mM benzamidine. The eluted single peak fraction was subjected to gel filtration chromatography (HiLoad 16/60 Superdex-75 (GE Healthcare) equilibrated in 150 mM HEPES pH 7.4 buffer, 100 mM NaCl, 1 mM β-mercaptoethanol, 0.2 % NaN_3_, 1 mM benzamidine, 0.1 mM PMSF. Use of the metalloprotease inhibitor EDTA was avoided in order to prevent chelation of divalent cations (e.g., Mg^2+^) in subsequent titration studies. The resulting fractions containing target protein were concentrated in an Amicon-15 centrifugal filter device (Millipore) to a final concentration of 38 mg/mL.

Uniformly ^15^N enriched CNNM4_cNMP-Ctail_ construct was similarly expressed in *E. coli* BL21 (DE3) grown in a modified auto-induction medium [59] with 2.5 g/L of ^15^NH_4_Cl as the only nitrogen source.

### 4.2. NMR Analysis

For our NMR titration studies we employed samples of U-^15^N labeled CNNM4_BAT_ after buffer exchange from conventional PBS (containing phosphate that may compete with ATP for Mg^2+^ complexation) to HEPES. While 50 mM HEPES was sufficient for the titrations with MgCl_2_, we increased its concentration to 100 mM for titrations with ADPNP to compensate for any pH decrease from hydrolysis of its phosphate moieties. The preset pH 7.4 was verified at the end points of titration using a pH electrode while the very pH sensitive ^1^H NMR signals of HEPES (at 3.77, 3.08, 2.91, and 2.85 ppm) allowed to directly monitor the pH stability in situ. Thus, we confirmed that the pH did not drop by more than 0.1 units during titrations. The NMR samples furthermore contained 100 mM NaCl, 0.02% (*w*/*v*) NaN_3_, 1 mM benzamidine, and 0.1 mM PMSF to increase their long-term stability. All experiments were measured at 298 K on an 800 MHz BRUKER AVANCE III spectrometer equipped with a TCI cryoprobe. For each titration point we recorded a 1D ^1^H NMR spectrum (with water suppression by excitation sculpting) to monitor the pH on the HEPES signals and the amount of ADPNP added, and a high-resolution fast 2D ^15^N-HSQC (with water suppression by excitation sculpting and broad-band ^1^H polarization flip-back) to monitor ligand induced shifts of protein amide signals. We also confirmed ADPNP binding by recording ^1^H_protein_→^1^H_ADPNP_ STD spectra (saturation transfer difference, measured in fully interleaved mode with alternating selective saturation of protein methyl resonances at 0.76 ppm during 1 to 3 s, a 100 ms T_2_ filter to suppress residual protein signal, and 3 s interscan recovery delay to avoid subtraction artifacts).

NMR titration experiments were similarly measured for U-^15^N labeled CNNM4_cNMP-Ctail_ dissolved in a regular PBS buffer (pH 7.4, 5 % ^2^H_2_O, 0.01% NaN_3_, 20 µM DSS) since Mg^2+^ binding to this domain was not studied. Instead, we added up to an eightfold excess of cAMP or cGMP (800 to 100 µM CNNM4_cNMP-Ctail_) to test their possible binding, but could not detect any significant signal shifts in superpositions of the 2D ^15^N HSQC spectra of CNNM4_cNMP-Ctail_ in the absence and presence of cNMP. TopSpin (Bruker) was used for NMR data processing and analysis. Chemical shifts were measured relative to internal DSS for ^1^H and calculated for ^15^N [60].

### 4.3. Size Exclusion Chromatography with Multiangle Light Scattering (SEC-MALS)

Static light scattering experiments were performed at 25 °C with a DAWN-HELEOS light scattering detector and Optilab rEX differential refractive index detector (Wyatt Technology, Santa Barbara, CA, USA) attached to a Superdex 200 10/300 GL column (GE HealthCare, Madrid, Spain). The column was equilibrated with running buffer (20 mM HEPES pH 7.4, 200 mM NaCl, 1 mM TCEP, 0.1 μm filtered) and the SEC-MALS system was calibrated with a 1 g/L BSA sample in the same buffer. Aliquots of 100 μL of each protein sample and at each concentration (1 and 10 g/L in the running buffer) were then injected into the column at a flow rate of 0.5 mL/min. ASTRA software (Wyatt Technology) was used for data acquisition and analysis. Based on repeated measurements with the BSA reference samples we estimated a 5% experimental error in the derived molar masses.

### 4.4. Isothermal Titration Calorimetry (ITC)

ITC experiments were performed with a Low-Volume NanoITC calorimeter (TA-Waters) using 50 μM protein solutions as analyte, except in titrations with Mg-ATP where the protein concentration was 40 μM. The titrant stock solutions were 10–13 times more concentrated than the analyte concentration to assure final titrant-to-analyte molar ratios above 3. Data were processed with Nanoanalyze (TA-Waters) and represented with Origin 2018 (Originlab, Northampton, MA, USA). The absence of any saturation behaviour in the thermograms made fitting unnecessary.

### 4.5. Small Angle X-Ray Scattering (SAXS)

SAXS data were collected in the bioSAXS beamline B21 at the Diamond Light Source (Harwell, UK) for the constructs CNNM4_cNMP_, CNNM4_BAT-cNMP-Ctail_, and the PRL-1/CNNM4_BAT-cNMP-Ctail_ complex. The latter two samples were analyzed in both absence and presence of MgATP (5 mM). The protein samples (46 µL, 5 mg/mL) were injected onto a KW-403 size-exclusion column at 20 °C in 20 mM HEPES buffer pH 7.4, 200 mM NaCl, 1 mM DTT and the output from the Agilent HPLC was directed through a 1.6 mm diameter quartz capillary cell under vacuum at a flow rate of 0.08 mL/min. A total of 620 frames (3 s exposure time) were collected with a PILATUS 2M (Dectris, Switzerland) detector at a distance of 4.014 m from the sample; the two-dimensional images were subsequently corrected for variations in beam current, normalized for exposure time, and processed into 1D scattering curves using GDA and DAWN software (Diamond Light Source, UK). The background was manually subtracted using ScÅtter (available online: http://www.bioisis.net/scatter, accessed on 10 December 2019). All collected SAXS data and experimental parameters are summarized in Table 2.

The interpolated 1D SAXS curves were initially analyzed to judge the data quality and obtain basic structural information related to the protein size and shape (Figure 4 and Figure 5). One structural parameter is the radius of gyration, R_g_, derived from the slope of the Guinier plot ln(Iq) vs q^2^ (q = 4πsin(θ)/λ is the scattering vector, 2θ the scattering angle, λ the wavelength). For globular proteins, this plot is expected to be linear at small q (i.e., q∙R_g_ < 1.3) and linearity of the Guinier plot is considered a quality measure of the data, but does not ensure ideality of the sample. SAXS curve analysis also provides an estimate of the protein’s molecular mass that, in turn, relates to its oligomeric state and approximately corresponds to half of its Porod volume (i.e., excluded volume of the hydrated protein), derived from the scattering intensity at larger q (Porod plot). Finally, the maximum size of a protein, D_max_, can be obtained by analyzing the SAXS data with a Pair-Distance Distribution Function, P(r), for all electrons in the protein. P(r) is obtained by indirect Fourier transformation [61] in a trial-and-error process, at the end of which the obtained D_max_ corresponds to the smoothest positive distribution. Changes in the D_max_ of a protein relate to conformational changes. It is also possible to calculate R_g_ from P(r) and compare its value with the one estimated from the Guinier plot. Low resolution structures derived from experimental scattering data were constructed by ab initio modelling using the program GASBOR [62] by aligning, averaging, and filtering 10 independently calculated dummy residue models (C-α models) [63]. All programs are included in ATSAS package [54]. The final SAXS models were deposited and are available at SASBDB [64] (corresponding access codes are listed in Table 2).

### 4.6. Protein Crystallization

The crystallizability of all protein constructs was evaluated with XtalPred [65]. CNNM4_BAT_ crystals were obtained as described previously [40] while crystals of SeMet labeled CNNM4_cNMP_ were grown by sitting drop vapor diffusion at 18 °C, mixing 0.5 μL protein (8 mg/mL) with 0.5 μL precipitant solution (0.5 M sodium formate, 5% (*w*/*v*) polyethylene glycol (PEG) 3350, 0.1 M Tris pH 7.2). All crystals were cryoprotected by immersion in mother liquor containing 20% (*v*/*v*) glycerol, and flash cooled in liquid nitrogen.

### 4.7. X-Ray Structure Determination and Refinement

All diffraction data were collected at the Diamond Light Source (Didcot, UK) beamline I03, and at the ALBA synchrotron (Barcelona, Spain) beamline BL13 (XALOC).

The anisotropy of the CNNM4_359–511_ diffraction data was corrected with the UCLA MBI Diffraction Anisotropy Server [66].

Diffraction intensities were indexed, integrated, scaled, and merged using XSCALE XDS [67]. Initial phases for CNNM4_359–511_ were determined by molecular replacement with Phaser [68], using CNNM2_BAT_ (PDB ID 4IY4) [5] as search model. Non-crystallographic symmetry averaging over appropriate regions of independent monomers in the asymmetric units was applied. The crystal structure of SeMet labeled CNNM4_545–730_ was solved by single wavelength anomalous dispersion (SAD) for phase determination (Table 1) after allocating the selenium atoms for CNNM4_545–730_ with SHELXD [69]. Initial phases and density modification used Autosol in PHENIX [70] and an initial model was built with BUCCANEER [71]. Refinement with PHENIX and/or REFMAC5 [72] was alternated with manual modeling by COOT [73]. The geometric quality of the models was assessed with MolProbity [74]. All crystal characteristics and final refinement statistics are summarized in Table 1. Figures showing 3D protein structures were prepared with PyMOL (available online: http://www.pymol.org/, accessed on 10 December 2019) and Chimera [56]. For surface calculation we employed the PISA server [75].

## Figures and Tables

**Table 1:** Data statistics and refinement. One crystal was used per data set. Values in parentheses refer to the high resolution shell.

Proteins	CNNM4_BAT_	CNNM4_cNMP_
**Data Collection and Process**		
Beamline	ESRF, ID14-1	DIAMOND, I03
Radiation wavelength (Å)	0.934	0.9794
Space group/PDB ID	*C2*/6RS2	*P3221*/6G52
a (Å) b (Å) c (Å)	91.39 141.36 87.89	116.74 116.74 243.46
Molecules per a.u.	4	9
Resolution (Å)	43.94–3.69 (3.76–3.69)	243.46–3.69 (3.99–3.69)
R_sym_^a^	0.047 (0.437)	0.179 (1.661)
R_meas_^b^	0.052 (0.485)	0.184 (1.707)
R_pim_^c^	0.023 (0.209)	0.042 (0.387)
No. of observations	62,620	413,463
No. of unique reflections	11,864	21,343
Mean I/I	21 (3.4)	13.9 (2.8)
CC1/2	0.99 (0.91)	0.99 (0.87)
Completeness (%)	98.7 (90.7)	99.7 (98.5)
Redundancy	5.3 (5.2)	19.4 (19.3)
Mosaicity (°)	0.2	0.1
**Refinement Statistics**		
No. of working/test reflections	44,826/1178	20,676/1989
R_work_^d^/R_free_^e^	0.23/0.28	0.2844/0.3026
No. of atoms		
Protein	4567	9252
Ligand	-	-
Water	-	-
Average B factors (Å^2^)		
Protein	105,94	100
Ligand	-	-
Water	-	-
RMSDs		
Bond lengths (Å)/angles (°)	0.004/0.683	0.003/0.748
Ramachandran plot statistics (%)		
Residues in most favored regions	97.5	99
Residues in additionally allowed regions	2.5	1
Residues in disallowed regions	0	0

One crystal was used per data set. Values in parentheses are for the highest resolution shell. R_sym_^a^ = Σ_hkl_ Σ_i_ |I_i_ (hkl) − <I(hkl)>I/Σ_hkl_ Σ_i_ I_i_(hkl); R_meas_^b^ = Σ_hkl_ Σ_i_ |I_i_ (hkl) − <I(hkl)>I/Σ_hkl_ Σi I_i_(hkl); ^c^ R_pim_= R_pim_ = Σ_hkl_ Σ_i_ |I_i_ (hkl) − <I(hkl)>I/Σ_hkl_ Σ_i_ I_i_(hkl). R_work_^d^ = Σ |F_o_ − F_c_|/ΣF_o_. R_free_^e^ = Σ |Fo − Fc|/ΣF_o_, calculated using a random 5 % of reflections that were not included throughout refinement. FMT = formate. N/A= Not applicable.

**Table 2:** Data collection and structural parameters by SAXS.

**Data Collection Parameters**					
Beamline	B21, Diamond Light Source, Harwell (UK)				
Detector	Pilatus 2M				
Beam size	0.2 × 0.2 mm				
Energy	12.4 keV				
Sample-to-detector distance (mm)	4014				
*q* range (A^−1^)	0.0038–0.42				
Exposure time (s)	3				
Number of frames	620				
Temperature (K)	293				
Mode	SEC online				
**Structural Parameters**					
Protein construct	**CNNM4_cNMP_**	**CNNM4_BAT-cNMP-Ctail_**	**CNNM4_BAT-cNMP-Ctail_ + MgATP**	**CNNM4_BAT-cNMP-Ctail_ + PRL-1**	**CNNM4_BAT-cNMP-Ctail_ + MgATP + PRL-1**
SASBDB access code	SASDER8	SASDEQ8	SASDES8	SASDEP8	SASDEN8
Concentration range (mg/mL)	7.0	5.0	4.7	8.5	5.0
*q* Interval for Fourier inversion (Å^−1^)	0.014–0.2018	0.007–0.164	0.010–0.109	0.09–0.173	0.009–0.125
*R_g_* [from P(r)] (Å)	25.67 ± 1.32	41.62 ± 2.12	39.15 ± 1.38	47.90 ± 5.24	47.20 ± 2.55
*R_g_* [from Guiner approximation] (Å)	25.52 ± 1.65	40.22 ± 0.54	39.23 ± 1.98	46.55 ± 0.81	44.48 ± 1.42
*sR_g_* limits [from Guiner approx.]	0.34–1.30	0.31–1.30	0.41–1.30	0.36–1.29	0.38–1.29
D_max_ (Å)	96	166	143	185	183
Porod volume estimate (nm^3^)	102	156	149	226	216
Porod exponent	2.3	3.2	3.3	3.1	3.1
Molecular Mass (kDa)					
from Porod volume (×0.58)	59	90	86	131	125
from amino acid sequence	21	48	48	68	68
**Software Employed**					
Primary data reduction	DAWN pipeline (Diamond Light Source, UK)				
Data processing	ScÅtter v3.1v				
Ab initio modelling	GASBOR				
Validation and averaging	DAMAVER/DAMCLUST				
Computation of model intensities	CRYSOL/CORAL				

q = 4πsin(θ)/λ, where 2θ is the scattering angle and λ the wavelength.

## Supplementary Materials

Supplementary materials can be found at https://www.mdpi.com/1422-0067/20/24/6279/s1.
