# Supplementary material for: Structural Insights into the Intracellular Region of the Human Magnesium Transport Mediator CNNM4
Source: Int J Mol Sci. 2019 Dec 12;20(24):6279. doi: 10.3390/ijms20246279 (PMC6940986; doi:10.3390/ijms20246279)
Supplement: Supplementary file 1 [file ijms-20-06279-s001.pdf]

Supplementary Information

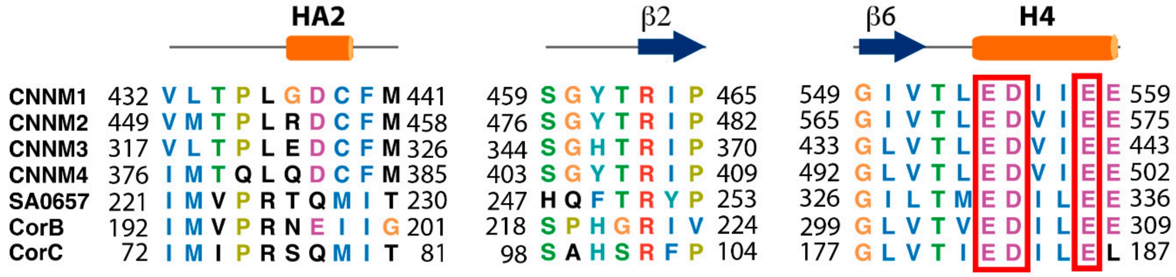

Supplementary Figure S1. Sequence alignment of the three structural blocks that conforms site S2 in the CNNMs and in bacterial proteins SA0657, CorB and CorC (Uniprot codes A0A0H3JL60, A0A0H3NER8 and P0A2L3 respectively). The conserved acidic cluster (which prevents binding of ATP in the absence of Mg^{2+} in CNNMs [5]) is highlighted with a red rectangle. Secondary structure elements are represented above the alignment.

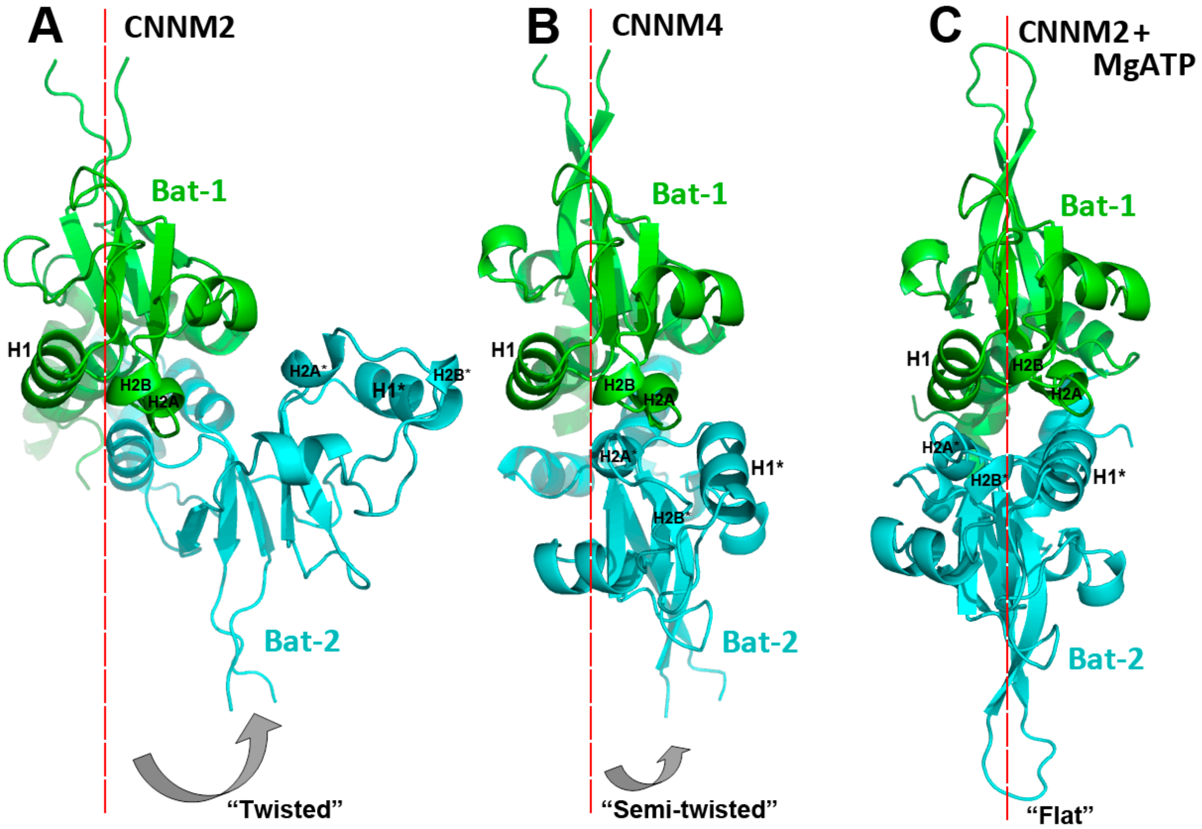

Supplementary Figure S2. **Structure of the CBS module in CNNMs in three distinct conformations.** The two Bateman modules are shown in *cyan* and *green*. CNNM2 adopts two limiting conformations: (**A**) a *twisted* disk in the absence of MgATP, and (**C**) a *flat* disk in the presence of bound MgATP. The plane intersecting the central β-sheets of one CBS module is represented by a *red* line to emphasise the *flat* to *twisted* conformational transition. (**B**) In the crystals, CNNM4 forms a *semi-twisted* disk in the absence of MgATP as an intermediate between conformations (**A**) and (**C**)

A

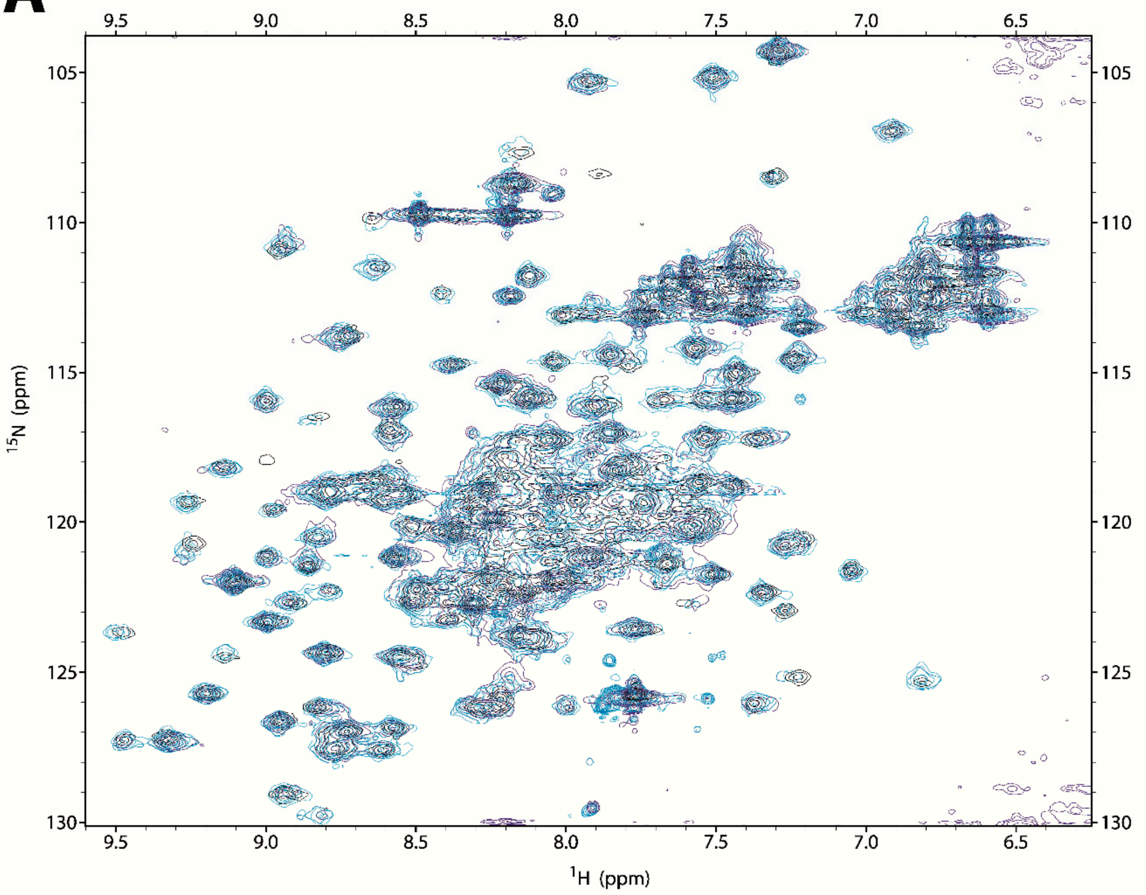

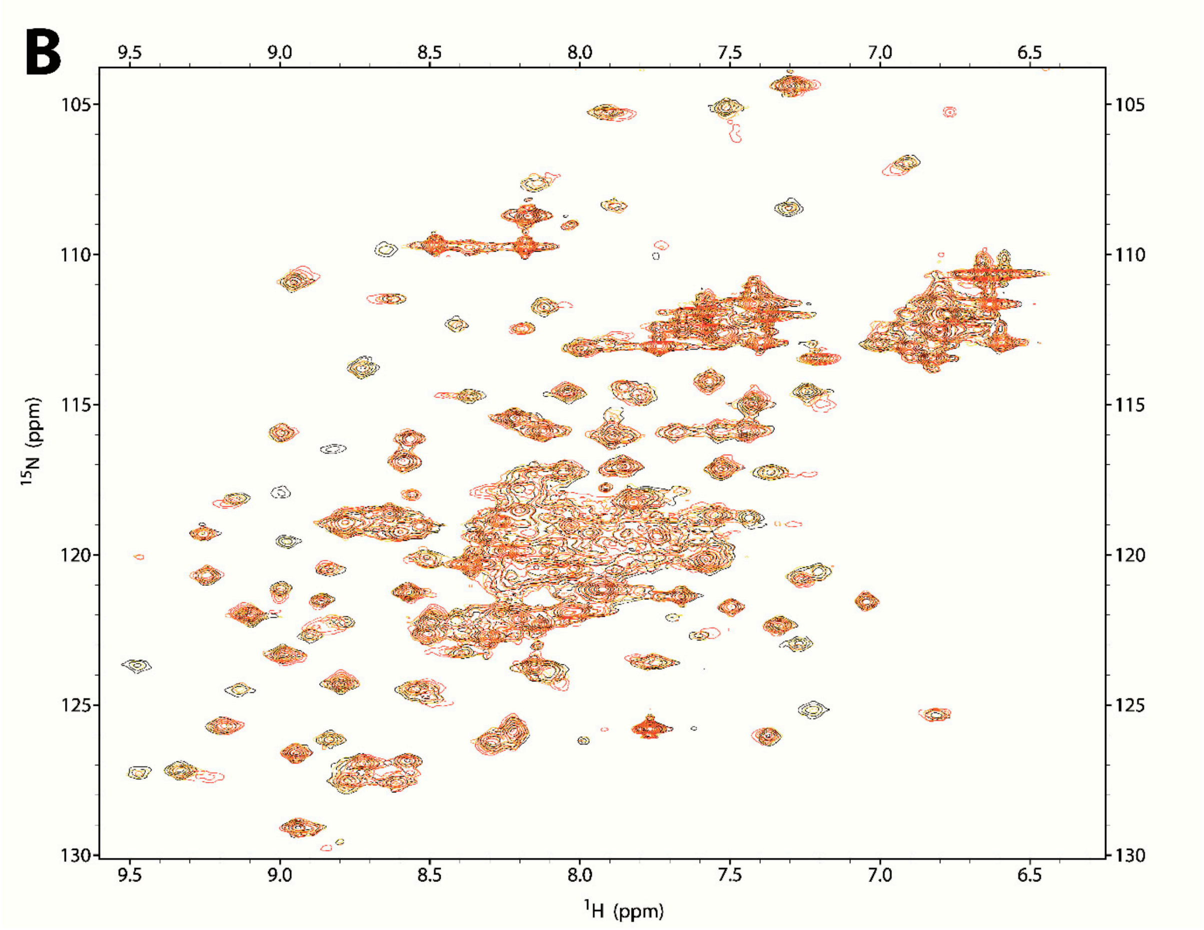

B
9.5
9.0
8.5
8.0
7.5
7.0
6.5
105
110
115
120
125
130
^{15}N (ppm)
^{1}H (ppm)

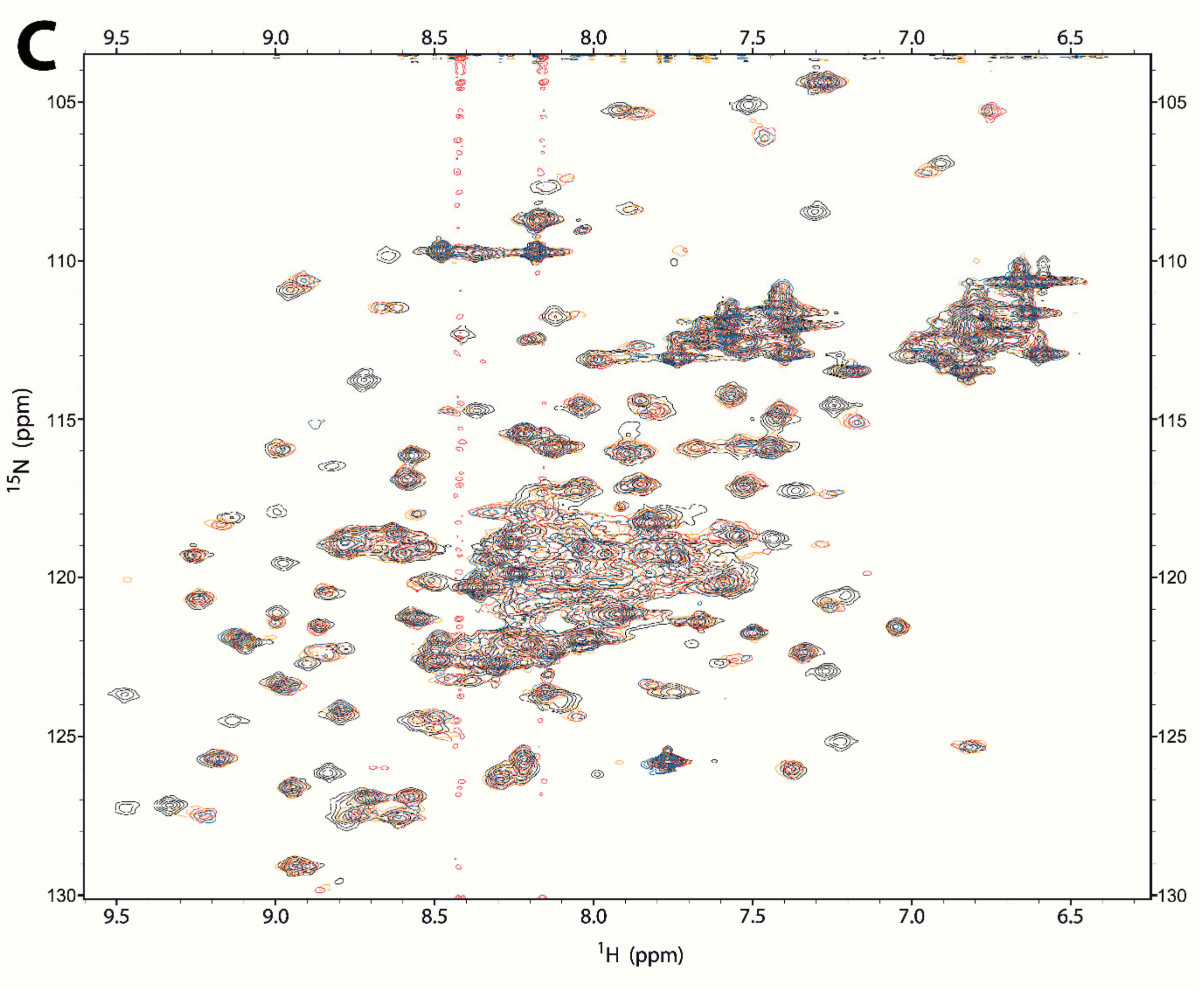
C
9.5
9.0
8.5
8.0
7.5
7.0
6.5
105
110
115
120
125
130
15N (ppm)
1H (ppm)

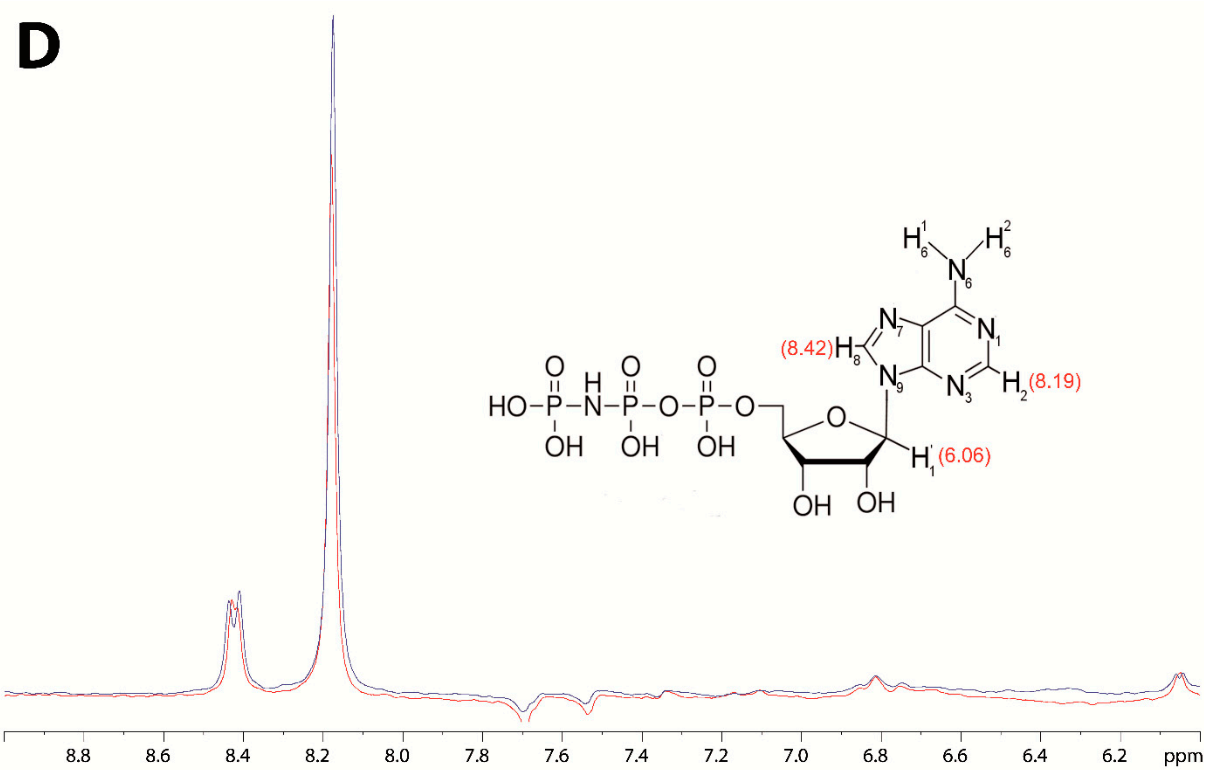

Supplementary Figure S3. (A) Ligand binding by hCNNM4BAT. (**A – C**) Observation via 2D ^{15}N HSQC NMR spectra of the protein (100 µM). The complete spectral region is shown (corresponding zoom regions in Figure 2). **A)** Effect of Mg^{2+} addition to a final concentration of 0 mM (*black*), 10 mM (*light blue*), 20 mM (*blue*), and 40 mM (*dark blue*). **(B)** Effect of ADPNP addition to a final concentration of 0 mM (*black*), 570 µM (*yellow*), 1.14 mM (*orange*), and 5.7 mM (*red*). **(C)** Effect of combined Mg^{2+} and ADPNP addition: 0 mM Mg^{2+} plus 0 mM ADPNP (*black*), 0 mM Mg^{2+} plus 5.7 mM ADPNP (*orange*), 0 mM Mg^{2+} plus 18 mM ADPNP (*red*), and 10 mM Mg^{2+} plus 5.7 mM ADPNP (*blue*). Spurious t_1 noise at ^{1}H frequencies of ca. 8.2 ppm and 8.4 ppm derive from protons H2 and H8 in the purine ring of ADPNP. (**D**) Observation via 1D $^1H_{protein}\rightarrow{}^1H_{ligand}$ Saturation Transfer Difference (STD) spectra of the ligand ADPNP (5.7 mM; 300 µM hCNNM4BAT). The superposed STD spectra in the absence (*red*) and presence (*black*) of Mg^{2+} (10 mM $MgCl_2$) confirm ADPNP binding in both cases, where the stronger STD signals in the presence of Mg^{2+} indicate positive cooperativity of Mg^{2+} and ADPNP binding. The molecular structure and assigned ^{1}H signals of ADPNP are shown in the inset.

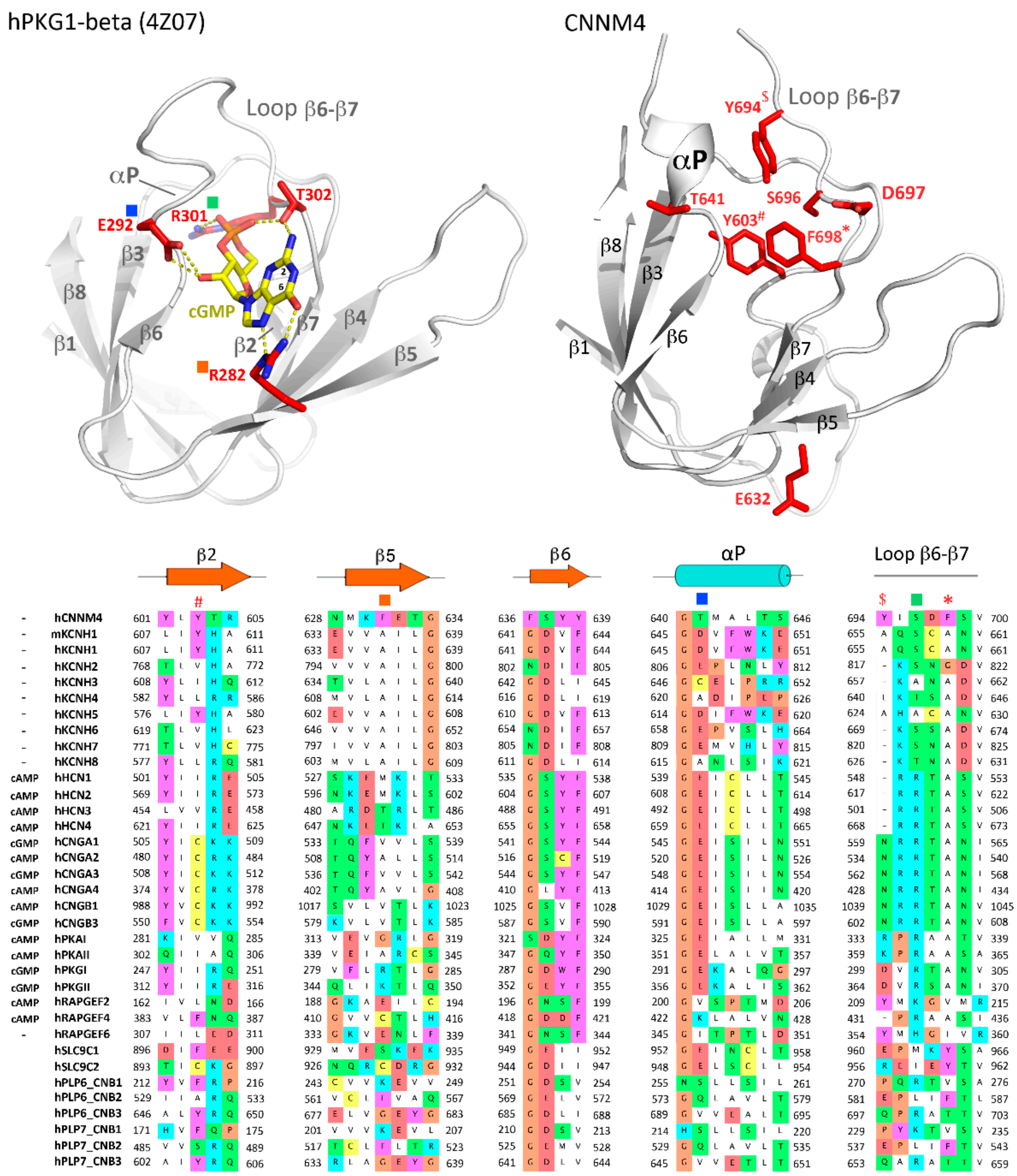

Supplementary Figure S4: Comparison of CNNM4cNMP with further CNBD or CNBDH domains. (*Top*) Ribbon and stick representation of the β-roll in the CNB domain of PKG-1 (*left*; PDB ID 4Z07) and CNNM4cNMP (*right*; PDB ID 6G52). Residues involved in cGMP (*yellow*) interaction are highlighted in *red*. CNNM4cNMP neither conserves these residues nor provides for sufficient space to host a cNMP ligand since the cavity is occupied by bulky residues. CNNM4 residues T641, S696, D697, and E632 are located at positions equivalent to PKG-1 residues E292, R301, T302, and R282, respectively (*Bottom*). Sequence alignment of *h*CNNM4 *vs* human CNBD and CNBDH containing proteins. The location of key residues involved in cNMP binding and their analogs in CNNM4cNMP are highlighted by colored symbols above the alignment (below the secondary structure elements). The known cNMP ligands are indicated in the first column, where known absence of cNMP binding is marked by (-). The Uniprot codes for the analyzed proteins are: *h*CNNM4, Q6P4Q7; *Mm*KCNH1, Q60603; *h*KCNH1, O95259; *h*KCNH2, Q12809; *h*KCNH3, Q9ULD8; *h*KCNH4, Q9UQ05; *h*KCNH5, Q8NCM2; *h*KCNH6, Q9H252; *h*KCNH7, Q9NS40; *h*KCNH8, Q96L42; *h*HCN1, O60741; *h*HCN2, Q9UL51; *h*HCN3, Q9P1Z3; *h*HCN4, Q9Y3Q4; *h*CNGA1, P29973; *h*CNGA2, Q16280; *h*CNGA3, Q16281; *h*CNGA4, Q8IV77; *h*CNGB1, Q14028; *h*CNGB3, Q9NQW8; *h*PKAI, P10644; *h*PKAII, P31323; *h*PKGI, Q13976; *h*PKGII, Q13237; *h*RAPGEF2, Q9Y4G8; *h*RAPGEF4, Q9EQZ6; *h*RAPGEF6, Q8TEU7; *h*SLC9C1, Q4G0N8; *h*SLC9C2, Q5TAH2; *h*PLP6, Q8IY17; *h*PLP7, Q6ZV29

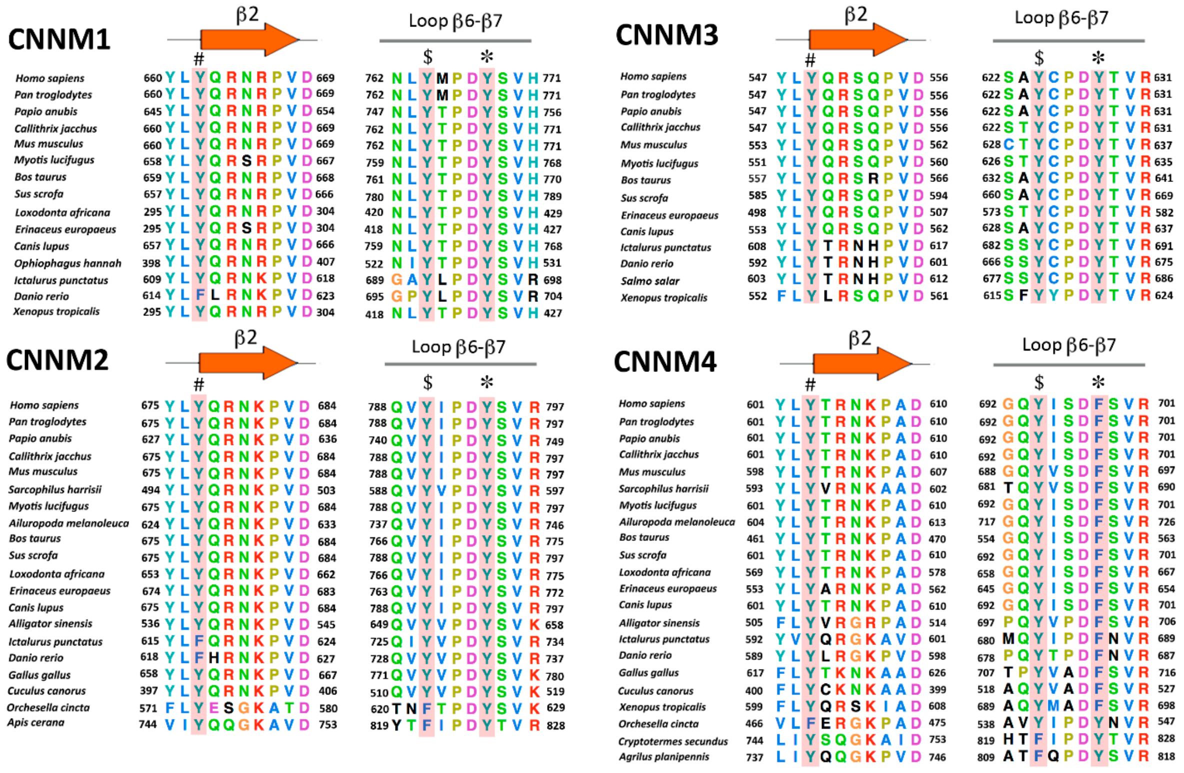

Supplementary Figure S5: **Sequence alignment of CNNM proteins from different species**. The indicated bulky hydrophobic residues Y603 (#), Y694 ($) and F698 (*) that occupy the main cavity of the β-roll in CNNM4cNMP are highly conserved in all CNNMs from different species, from insects to mammals, while not all organisms encode all four CNNM members. Residue numbers and secondary elements are indicated. The Uniprot codes for the analyzed proteins are: *Hs*CNNM1, Q9NRU3; *Pt*CNNM1, H2Q2E3; *Pa*CNNM1, A0A096P643; *Cj*CNNM1, F7I103; *Mm*CNNM1, Q0GA42; *Ml*CNNM1, G1P8Z7; *Bt*CNNM1, F1MD84; *Ss*CNNM1, F1S8W6; *La*CNNM1, G3TYT9; *Ee*CNNM1, A0A1S3AFW3; *Cl*CNNM1 F1PMJ7; *Oh*CNNM1, V8NPD9; *Ip*CNNM1, A0A2D0QLR5; *Dr*CNNM1, A0A0G2KKC2; *Xt*CNNM1, F6WMC5; *Hs*CNNM2, Q9H8M5; *Pt*CNNM2, K7CZV7; *Pa*CNNM2, A0A096P6G5; *Cj*CNNM2, U3E1C9; *Mm*CNNM2, Q3TWN3; *Sh*CNNM2, G3X2G0; *Ml*CNNM2, G1Q8B9; *Am*CNNM2, G1MHQ7; *Bt*CNNM2, E1BIL3; *Ss*CNNM2, A0A287AM25; *La*CNNM2, G3UIL2; *Ee*CNNM2, A0A1S3WKT5; *Cl*CNNM2, E2RJ19; *As*CNNM2, A0A1U7RW72; *Ip*CNNM2, A0A2D0QNG1; *Dr*CNNM2, A2ATX7; *Gg*CNNM2, A0A1D5PIB9; *Cc*CNNM2, A0A091G624; *Oc*CNNM2, A0A1D2NLQ6; *Ac*CNNM2, A0A2A3EE16; *Hs*CNNM3, Q8NE01; *Pt*CNNM3, A0A2I3TCA8; *Pa*CNNM3, A0A096NGS2; *Cj*CNNM3, U3DC74; *Mm*CNNM3, Q32NY4; *Ml*CNNM3, G1PS60; *Bt*CNNM3, F1N293; *Ss*CNNM3, F1STC7; *Ee*CNNM3, A0A1S3AJ95; *Cl*CNNM3, E2RGW9; *Ip*CNNM3, A0A2D0SQK4; *Dr*CNNM3, E7F3M2; *Ss*CNNM3, A0A1S3KYV5; *Xt*CNNM3, F7B785; *Hs*CNNM4, Q6P4Q7; *Pt*CNNM4, H2QID4; *Pa*CNNM4, A0A096NGR9; *Cj*CNNM4, F7I1N7; *Mm*CNNM4, Q69ZF7; *Sj*CNNM4, G3WZR3; *Ml*CNNM4, G1QFN; *Am*CNNM4, G1LFK1; *Bt*CNNM4, F1MK24; *Ss*CNNM4, F1STC6; *La*CNNM4, G3TEW1; *Ee*CNNM4, A0A1S3AIV6; *Cl*CNNM4, F1PJ05; *As*CNNM4, A0A1U7S4Z7; *Ip*CNNM4, W5UIQ1; *Dr*CNNM4, F1Q7I7; *Gg*CNNM4, A0A1D5P3M6; *Cc*CNNM4, A0A091H8N3; *Xt*CNNM4, A0JPA0; *Oc*CNNM4, A0A1D2NLL8; *Cs*CNNM4, A0A2J7RPU8; *Ap*CNNM4, A0A1W4XF17.

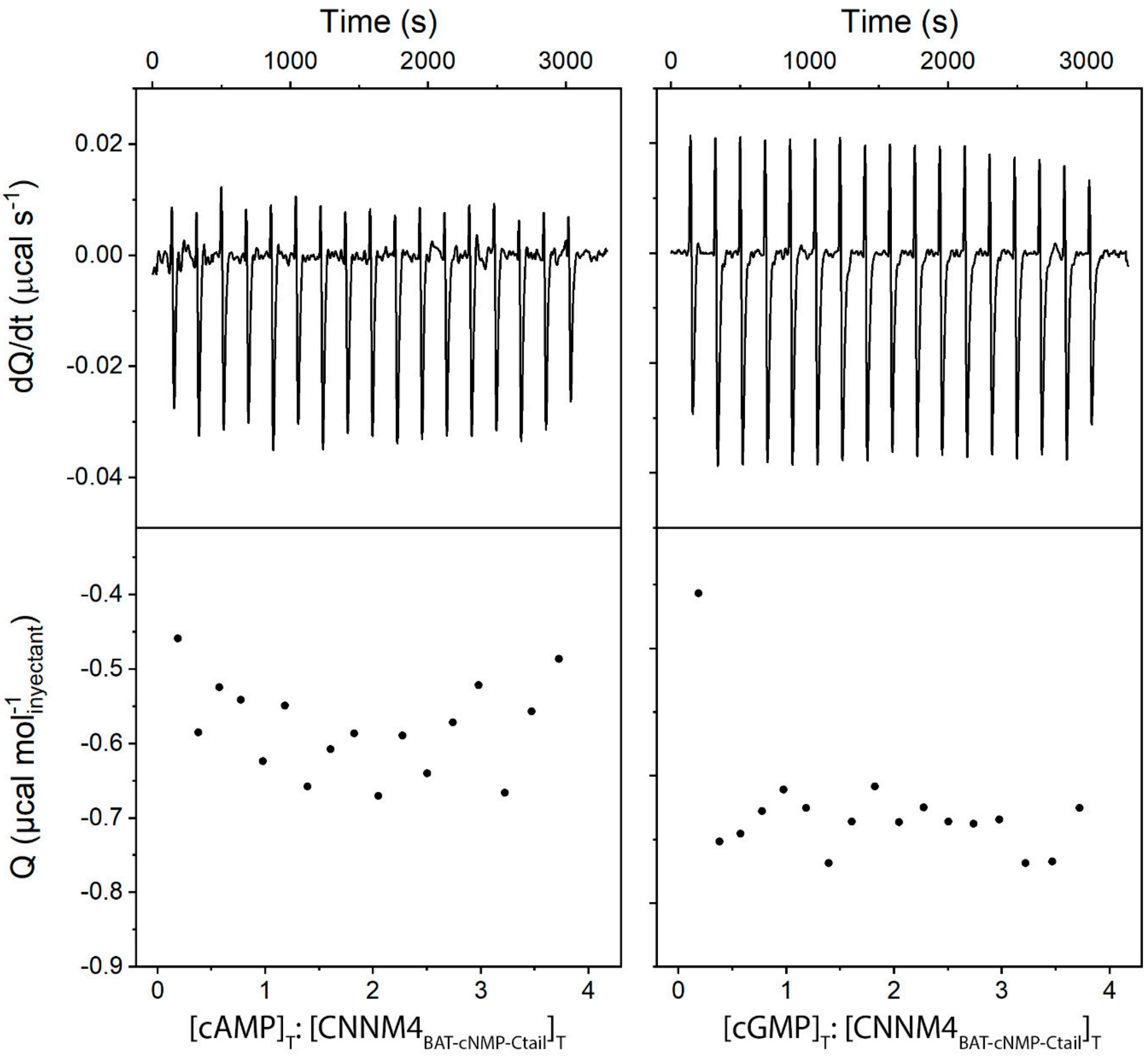

Supplementary Figure S6. **ITC data from cAMP (left) or cGMP (right) titration to CNNM4$_{\mathbf{BATcNMP\text{-}CTail}}$.** Upper panels show the thermogram, lower panels the mixing isotherm.

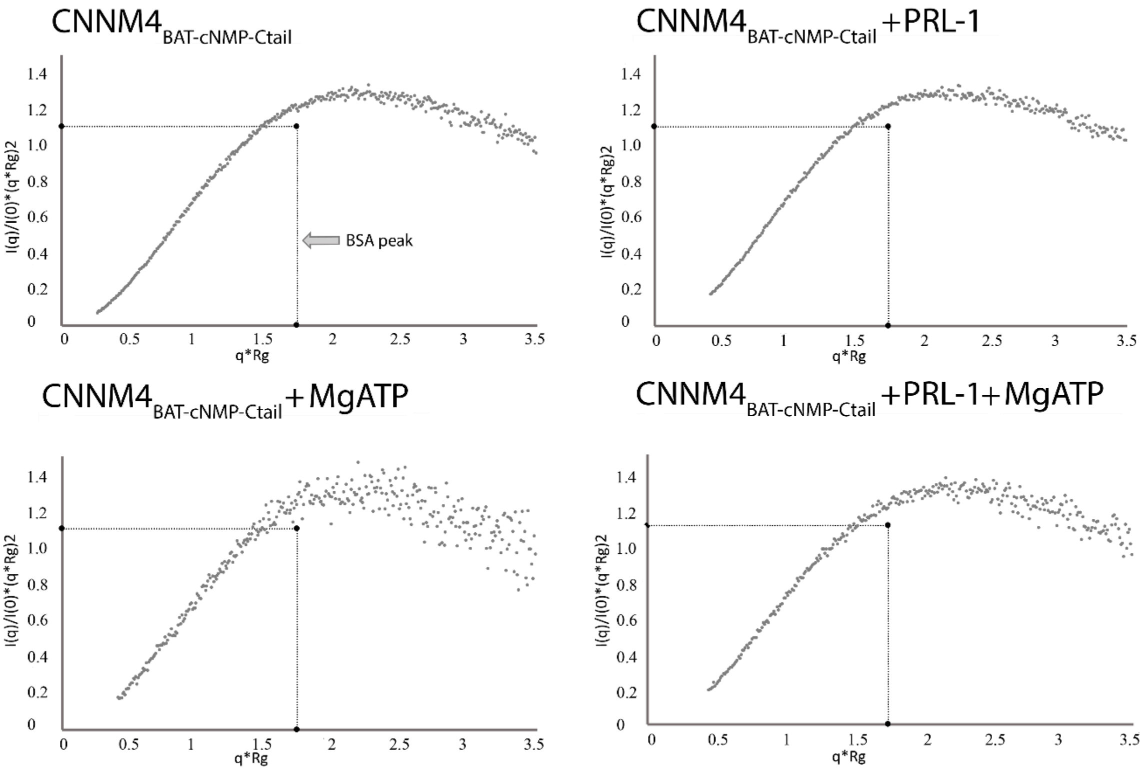

Supplementary Figure S7. Dimensionless Kratky plots of SAXS data for $CNNM4_{BAT\text{-}cNMP\text{-}Ctail}$ (*left*) in the absence (*above*) and presence (*below*) of MgATP, and for its complex with PRL-1 (*right*). In all cases, the plots indicate a well folded and elongated molecule. The pertaining BSA reference peak is indicated by a black dot.

Supplementary Movie S1: SAXS derived solution structure of CNNM4$_{BAT\text{-}cNMP\text{-}Ctail}$ in the absence of MgATP. The associated Bateman modules are shown in *orange* and *red*, their connected cNMP binding domains in *dark* and *light blue*, and the interdomain linkers (added with CORAL) in *dark* and *light green*, respectively. In the absence of ATP, the Bateman modules associate to form a *twisted* disk shaped CBS module. The two main cavities formed between connected CBS1 and CBS2 motives are named S1 and S2, where S2 provides the MgATP binding site. The CBS module is located above the cNMP domain dimer, with their CBS1 motives inserting into the main cleft formed between both cNMP monomers. Helix H0 links the Bateman module to the preceding DUF21 transmembrane region at the distal end. The disordered αterminal tail (following the cNMP binding domain) is not shown.

Supplementary Movie S2: SAXS derived solution structure of the complete intracellular region, CNNM4$_{BAT\text{-}cNMP\text{-}Ctail}$, in the presence of MgATP. Naming and colouring as in movie S1. MgATP binding causes the Bateman modules to shift into a *flat* conformation of the CBS module by sliding of their CBS1 ends within the cleft formed by the cNMP domain dimer.

Supplementary Movie S3: SAXS derived solution structure of the complex formed by CNNM4$_{BAT\text{-}cNMP\text{-}Ctail}$ with PRL-1 in the presence of MgATP (indicated by a label). Naming and colouring as for movie S1, with PRL-1 shown in *yellow*. The location of the long loop of the cNMP domain (not visible in the crystals) is indicated.

Supplementary Movie S4. Model of the twisted-to-flat conformational change induced by MgATP in CNNM4. Binding of MgATP at the Bateman modules (colored in *red* and *orange*) triggers a rotation of their CBS motifs that transforms the disk shaped CBS module from a twisted to a flat conformation. The CBS1 motifs from the Bateman modules insert into the large cleft formed between the rigidly dimerized cNMP domains (in *dark* and *light blue*, respectively), which restricts their sliding during the conformational change. The linkers connecting both intracellular domains as well as the C-tail following the cNMP domain are omitted for clarity.
